# HDAC1/2/3 are major histone desuccinylases critical for promoter desuccinylation

**DOI:** 10.1038/s41421-023-00573-9

**Published:** 2023-08-15

**Authors:** Jialun Li, Lu Lu, Lingling Liu, Xuelian Ren, Jiwei Chen, Xingzhi Yin, Yanhui Xiao, Jiwen Li, Gang Wei, He Huang, Wei Wei, Jiemin Wong

**Affiliations:** 1 Wuhu Hospital, East China Normal University, Wuhu, Anhui China; 2 Shanghai Key Laboratory of Regulatory Biology, Institute of Biomedical Sciences and School of Life Sciences, East China Normal University, Shanghai, China; 3 CAS Key Laboratory of Computational Biology, Shanghai Institute of Nutrition and Health, University of Chinese Academy of Sciences, Chinese Academy of Sciences, Shanghai, China; 4 Shanghai Institute of Materia Medica, Chinese Academy of Sciences, Shanghai, China; 5 Institutes of Biomedical Sciences, Fudan University, Shanghai, China

**Keywords:** Acetylation, Transcription

## Abstract

Lysine succinylation is one of the major post-translational modifications occurring on histones and is believed to have significant roles in regulating chromatin structure and function. Currently, histone desuccinylation is widely believed to be catalyzed by members of the SIRT family deacetylases. Here, we report that histone desuccinylation is in fact primarily catalyzed by the class I HDAC1/2/3. Inhibition or depletion of HDAC1/2/3 resulted in a marked increase of global histone succinylation, whereas ectopic expression of *HDAC1/2/3* but not their deacetylase inactive mutants downregulated global histone succinylation. We demonstrated that the class I HDAC1/2/3 complexes have robust histone desuccinylase activity in vitro. Genomic landscape analysis revealed that histone succinylation is highly enriched at gene promoters and inhibition of HDAC activity results in marked elevation of promoter histone succinylation. Furthermore, our integrated analysis revealed that promoter histone succinylation positively correlates with gene transcriptional activity. Collectively, we demonstrate that the class I HDAC1/2/3 but not the SIRT family proteins are the major histone desuccinylases particularly important for promoter histone desuccinylation. Our study thus sheds new light on the role of histone succinylation in transcriptional regulation.

## Introduction

Lysine succinylation (Ksu) is the addition of a succinyl group on the epsilon-amino group of lysine. As a member of recently emerging lysine acylations that include propionylation, butyrylation, crotonylation, etc., Ksu has been identified in a wide range of proteins from prokaryotes to human beings^1,2^. Ksu requires donor succinyl-CoA and occurs in an enzymatic or non-enzymatic manner^3,4^. Although Ksu is highly enriched in mitochondria in which the concentration of succinyl-CoA is relatively high^5,6^, succinyl-CoA is also abundant in the nuclear compartment^7^ and histones are well known for Ksu modification^8^. As succinylation not only neutralizes the positive charge of lysine but also brings in a negative charge and a bulky side chain, Ksu is believed to have stronger impacts on chromatin structure and function than acetylation and methylation^2,9,10^. In support of this idea, multiple in vitro biochemical studies have shown that Ksu robustly promotes DNA unwrapping, nucleosome instability, and transcriptional activation^11–14^.

While succinylation in mitochondria may be a non-enzymatic event^4^, a few histone acetyltransferases have been shown to catalyze site-specific Ksu on histones. For example, KAT2A and HAT1 have been shown to catalyze H3K79 and H3K122 succinylation, respectively^15,16^. In addition, CBP/p300 have also been shown to succinylate histones^12^. Functionally, histone succinylation has been shown to promote transcription, tumor cell proliferation, and tumor development^12,15,16^.

Like other lysine modifications, Ksu is also a dynamic modification^2,6,17^. The biological function of Ksu was poorly understood until the identification of SIRT5, a member of Sirtuin family NAD^+^-dependent deacetylases, as the first and the only major desuccinylase so far^5^. SIRT5 is also known for its lysine demalonylase and deglutarylase activities but has very weak deacetylase activity^5,18^. SIRT5 mainly resides in mitochondria. Consistently, loss of SIRT5 leads to hyper-succinylation of a variety of mitochondrial proteins, and many SIRT5 substrates are involved in metabolic pathways such as fatty acid metabolism and TCA cycle^19–21^. Primarily based on in vitro studies, SIRT5 has been implicated as the enzyme for histone desuccinylation. More recently, SIRT7 was reported to catalyze H3K122 desuccinylation^22^. However, whether the SIRT family deacetylases are responsible for bulk histone desuccinylation in vivo and how histone succinylation is dynamically regulated remain poorly understood.

In this study, we surprisingly found that the class I HDACs (HDAC1/2/3) rather than the SIRT family members are the major histone desuccinylases in vivo. Histone deacetylases in mammalians consist of two large families, the Zn^2+^-dependent HDAC family (HDAC 1–11)^23^ and the NAD^+^ dependent SIRT family (SIRT 1–7)^24^. The 11 HDACs are further categorized into class I (HDAC 1–3 and 8), class IIa (HDAC 4, 5, 7, and 9), class IIb (HDAC 6 and 10), and class IV (HDAC11)^25^. HDAC1/2/3 exist in large corepressor complexes and represent bulk HDAC activity in cells^26–31^. Recent studies from our and other groups have demonstrated that HDAC1/2/3 are also active for histone decrotonylation^32^, de-β-hydroxybutyrylation, and delactylation^33,34^. In contrast to the previous in vitro assays that all recombinant HDACs were inactive for histone desuccinylation, we showed that the mammalian HDAC1/2/3 complexes possess robust histone desuccinylase activity in vitro. We also presented evidence that HDAC8 lacks intrinsic histone desuccinylase activity in vitro. We further demonstrated that histone succinylation is highly enriched at the gene promoters and that promoter histone succinylation level correlates positively with transcription activity.

## Results

### HDACs but not SIRTs are responsible for bulk histone desuccinylation in cells

**Fig. 1:**
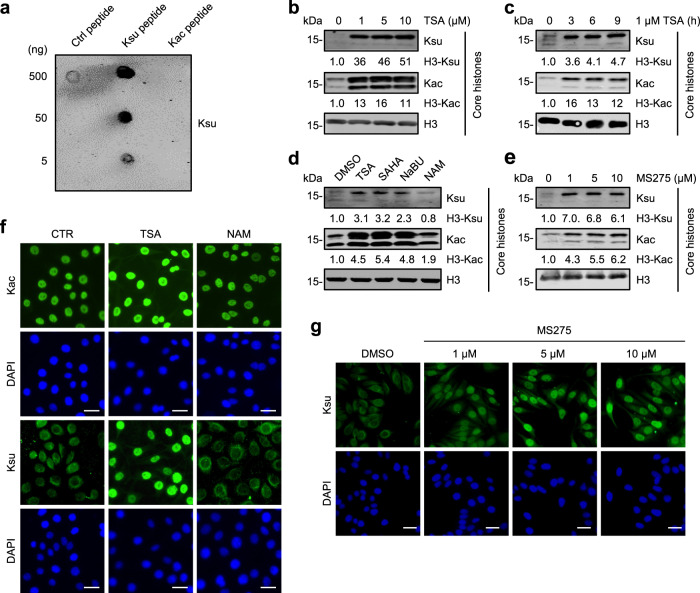
Marked elevation of histone succinylation by inhibition of HDACs but not Sirtuins. **a** Dot blot assay showing the specificity of a pan anti-Ksu antibody. Control peptide, unmodified H3 N-terminal 1–21 aa; Ksu peptide, H3 1–21 aa with succinylated K9, K14, and K18; Kac peptide, H3 1–21 aa with acetylated K9, K14, and K18. The amounts of peptides in dot blot were as indicated. **b**–**e** WB analyses showing the levels of histone succinylation and acetylation in HeLa cells treated with an increasing dose of TSA for 12 h (**b**), 1 μM TSA for different periods (**c**), different inhibitors (**d**), and different concentrations of the class I HDAC inhibitor MS275 (**e**). **f** IF assay showing the different effects of TSA and NAM treatment on cellular succinylation and acetylation levels. HeLa cells were treated with 1 μM TSA or 20 mM NAM for 24 h. Scale bars, 20 μm. **g** IF staining showing the effect of MS275 treatment on cellular succinylation level. HeLa cells were treated with different concentrations of MS275 for 12 h. Scale bars, 20 μm.

To assess whether SIRTs are responsible for dynamic histone succinylation in mammalian cells, we treated HeLa cells with an increasing dose of the pan-SIRT inhibitor nicotinamide (NAM) for 24 h followed by western blot (WB) analysis. However, using a commercial antibody that was raised against succinylated lysine peptides (Ksu) and its Ksu specificity was validated by dot blot analysis (Fig. 1a and Supplementary Fig. S1), we observed that inhibition of SIRTs by NAM treatment did not increase the level of histone succinylation (Supplementary Fig. S2a). Furthermore, while WB analysis revealed a broadly elevated level of succinylated proteins in SIRT5 knockout (KO) HeLa cells generated by CRISPR-Cas9 technology (Supplementary Fig. S2b), loss of SIRT5 did not increase histone succinylation level as revealed by WB using either the pan-Ksu antibody or antibodies against site-specific succinylated histones (Supplementary Fig. S2c). Together, these data indicate that the SIRT family deacetylases may not be the primary enzymes for histone desuccinylation in cells.

We next tested whether the HDAC family member(s) could mediate histone desuccinylation. We treated HeLa cells with an increasing concentration of Trichostatin A (TSA), a well characterized pan-HDAC inhibitor. Subsequent WB analysis revealed that TSA treatment resulted in not only a marked increase of histone acetylation, as expected, but also a parallel increase of histone succinylation (Fig. 1b). Furthermore, inhibition of HDACs by 1 μM TSA for 3 h was sufficient to substantially elevate both histone acetylation and succinylation levels (Fig. 1c), suggesting that TSA most likely elevates histone succinylation by directly inhibiting histone desuccinylation. The ability to elevate histone succinylation is not unique to TSA, because treatment with two other HDAC inhibitors, SAHA and sodium butyrate^35^, also increased histone succinylation to a similar extent to that of TSA (Fig. 1d). In contrast, NAM treatment under the same condition failed to increase histone succinylation (Fig. 1d), implying that histone desuccinylation is primarily conducted by the HDAC rather than the SIRT family deacetylases. We also treated HeLa cells with MS275, a HDAC1/2/3-selective inhibitor^36^. As shown in Fig. 1e, MS275 treatment resulted in a robust increase of both histone succinylation and acetylation, indicating that the class I HDAC1/2/3 are likely the major histone desuccinylases in cells.

We next compared the effect of TSA and NAM treatment on Ksu by immunofluorescent (IF) staining. The representative results in Fig. 1f showed that TSA treatment resulted in strong elevation of both acetylation and succinylation in the nucleus. However, NAM treatment increased succinylation only in the cytoplasm with a pattern resembling mitochondria, a phenotype consistent with inhibition of mitochondria SIRT5^20,21^. To further support that SIRT5 is not responsible for bulk histone desuccinylation in cells, we found that TSA treatment markedly elevated histone succinylation in the SIRT5-KO cells to the same level as the control cells (Supplementary Fig. S2d). We also confirmed by IF staining that inhibition of HDAC1/2/3 by MS275 resulted in elevated nuclear succinylation in an MS275 dose-dependent manner (Fig. 1g).

Altogether, these results raised the possibility that histone desuccinylation is primarily carried out by the HDAC but not the SIRT family deacetylases. Furthermore, among the HDAC family, HDAC1/2/3 may represent the major histone desuccinylase activity in cells.

### HDACs are likely responsible for bulk histone desuccinylation in various cells

**Fig. 2:**
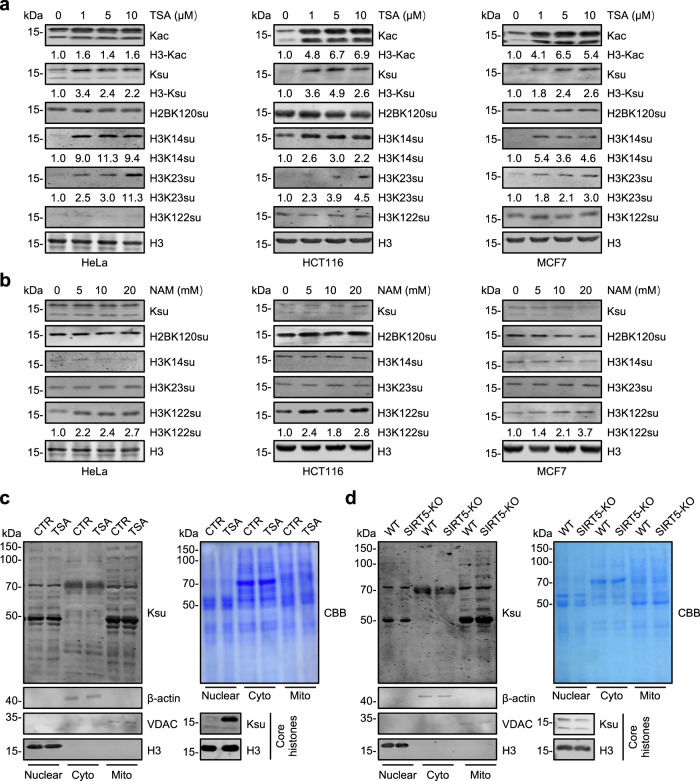
Inhibition of HDAC but not SIRT family deacetylases markedly elevates histone succinylation in various cells. **a**, **b** WB analysis of histone succinylation by pan-Ksu and site-specific histone Ksu antibodies. HeLa/HCT116/MCF7 cells were treated with different doses of HDAC inhibitor TSA for 12 h (**a**) or SIRT inhibitor NAM for 24 h (**b**). **c**, **d** WB analysis of succinylated proteins in nuclear, cytosolic, mitochondrial, and histone fractions derived from TSA-treated HeLa cells (**c**) or SIRT5 KO HeLa cells (**d**). TSA treatment: 1 μM TSA for 12 h. β-actin, H3, and VDAC as markers for cytosolic, nuclear and mitochondrial fractions, respectively.

To test whether HDACs are broadly responsible for histone desuccinylation in mammalian cells, we compared the effect of TSA treatment on histone succinylation in HeLa, colon cancer cell line HCT116, breast cancer cell line MCF7, and mouse embryonic stem cell line E14. As shown in Fig. 2a and Supplementary Fig. S3a, TSA treatment resulted in marked elevation of histone succinylation in all cells tested as shown by WB analysis using the pan-Ksu antibody. Furthermore, by using several commercially available site-specific histone succinylation antibodies, we found that TSA treatment markedly increased the succinylation levels of H3K14 (H3K14su) and H3K23 (H3K23su), but had little effect on succinylation of H2BK120 (H2BK120su) and H3K122 (H3K122succ) (Fig. 2a and Supplementary Fig. S3a). We confirmed by IF staining that TSA treatment markedly elevated the level of H3K23su but had no significant effect on H3K122su and H2B120su (Supplementary Fig. S3b). As expected^37^, TSA treatment impaired ES stemness activity (Supplementary Fig. S3c). Our result that TSA treatment has no effect on H3K122su is consistent with the previous report that SIRT7 has desuccinylase activity for H3K122^22^. Indeed, we found that while NAM treatment affected neither succinylation of bulk histones nor H3K14 and H3K23, it increased the level of H3K122su in all cell lines tested (Fig. 2b). Together, these results indicate that while SIRT family members may be responsible for histone desuccinylation at specific site(s) such as H3K122, HDACs are likely responsible for more sites and bulk histone desuccinylation.

Having observed that inhibition of HDACs resulted in bulk increase of histone succinylation, we next investigated whether HDACs also play a role in desuccinylation of non-histone proteins. Previous studies indicate that succinylated proteins are mainly mitochondrial and cytosolic proteins^1,38^; we therefore biochemically fractionated TSA-treated and -untreated cells into nuclear, cytoplasmic, and mitochondrial fractions and analyzed the succinylated proteins by WB analysis using pan-Ksu antibody. Interestingly, we found that TSA treatment, while leading to a substantial increase of histone succinylation, did not significantly affect protein succinylation in all three fractions (Fig. 2c). Furthermore, TSA treatment did not significantly alter the levels as well as the pattern of succinylation on non-histone proteins in whole-cell extracts (Supplementary Fig. S3d, e). On the contrary, the same analysis revealed that SIRT5-KO resulted in a marked increase of succinylation on mitochondrial proteins but had no effect on histone succinylation (Fig. 2d). Thus, the desuccinylase activity of HDACs appears to target mainly histones, whereas the SIRTs (especially SIRT5) are primarily responsible for the desuccinylation of mitochondrial proteins.

### HDAC 1/2/3 are the major histone desuccinylases in mammalian cells

**Fig. 3:**
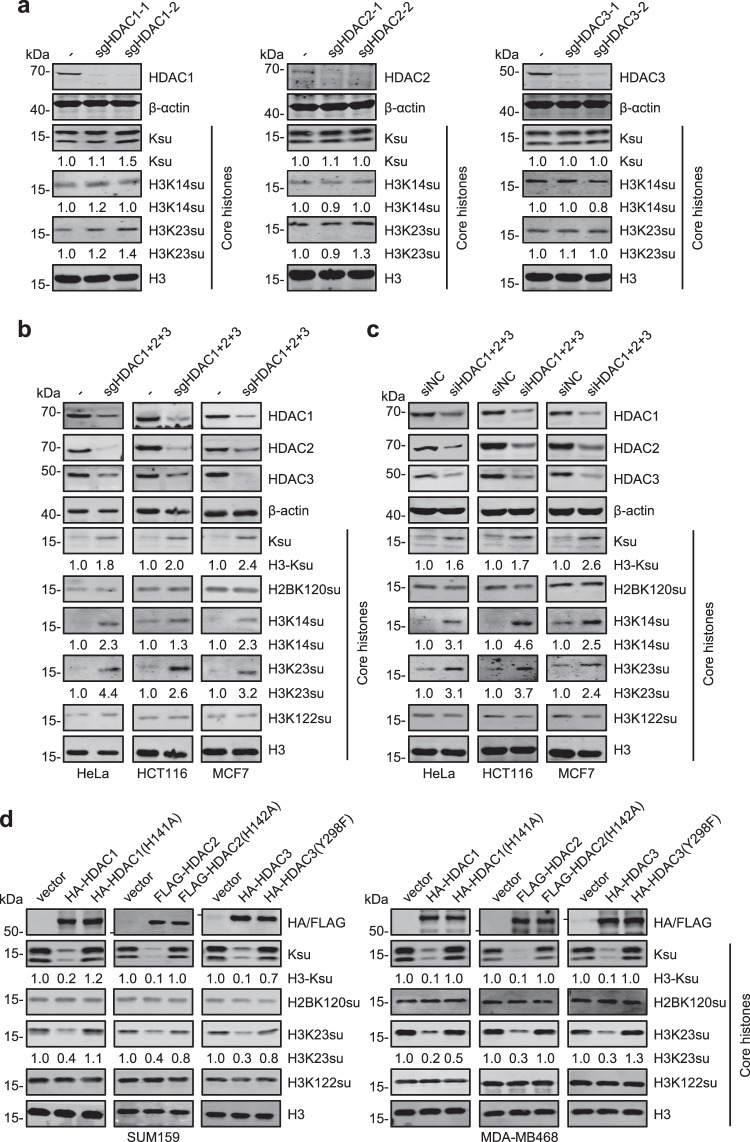
Class I HDAC 1/2/3 are the major histone desuccinylases in mammalian cells. **a** WB analysis showing the effect of single KO of *HDAC1*, *HDAC2*, or *HDAC3* on histone succinylation in HeLa cells. KO of each *HDAC* was conducted by two different sgRNAs. **b** WB analysis showing histone succinylation in HeLa, HCT116, and MCF7 cells with a combinatorial KO of *HDAC1/2/3*. **c** WB analysis showing histone succinylation in HeLa, HCT116, and MCF7 cells with combinatorial KD of *HDAC1/2/3* by siRNAs. Cells were treated with siRNAs for 72 h. **d** WB analysis showing the effect of ectopic expression of wild-type HDAC1, HDAC2, or HDAC3 and their corresponding HDAC activity-deficient mutants in two breast cancer cell lines SUM159 (left) and MDA-MB468 (right).

Having observed that MS275, a selective inhibitor for HDAC1/2/3, can effectively elevate histone succinylation as TSA, we next investigated the role of HDAC1/2/3 in histone desuccinylation. We first established individual KO of *HDAC1*, *HDAC2*, and *HDAC3* by CRISPR-Cas9 based gene disruption (Fig. 3a and Supplementary Fig. S4). WB analysis of core histone preparations revealed that KO of a single member of *HDAC1/2/3* did not result in elevation of histone succinylation (Fig. 3a), suggesting a redundant role for HDAC1/2/3 in histone desuccinylation. In agreement with this idea, simultaneous KO of *HDAC1/2/3* by CRISPR-Cas9 resulted in marked elevation of histone succinylation in all three cell lines, HeLa, HCT116, and MCF7, which we have tested (Fig. 3b). Using site-specific histone succinylation antibodies, we found that KO of *HDAC1/2/3* resulted in elevated levels of H3K14su and H3K23su and had no effect on H3K122su and H2B120su, consistent with the results of TSA treatment. To independently confirm this result, we simultaneously knocked down HDAC1/2/3 by using a mixture of siRNAs specifically targeting *HDAC1*, *HDAC2*, and *HDAC3*^32^. We confirmed by WB analysis that the siRNA treatment resulted in a more than 70% reduction of HDAC1, HDAC2, and HDAC3 proteins in all three cell lines (Fig. 3c). Notably, knockdown (KD) of HDAC1/2/3 by siRNA also resulted in a similar elevation of histone succinylation (Fig. 3c). These results therefore demonstrated that HDAC1/2/3 have redundant role in histone desuccinylation and likely represent the major histone desuccinylases in mammalian cells.

We next tested whether ectopically expressed HDAC1/2/3 could desuccinylate histones in cells. We noticed that most cell lines have a relatively low level of histone succinylation based on WB analysis. Interestingly, two breast cancer cell lines, SUM159 and MDA-MB468 possess a relatively higher level of succinylated histones. We then transfected these cells with HA- or FLAG-tagged wild-type HDAC1, HDAC2, HDAC3 and their HDAC activity-defective mutants (Fig. 3d). Subsequent WB analysis revealed that ectopic expression of either one of the wild-type HDAC1/2/3 could markedly downregulate the levels of global histone succinylation as well as H3K23su (Fig. 3d). Importantly, the same assay revealed that the HDAC1/2/3 mutants defective in HDAC activity were inactive in histone desuccinylation (Fig. 3d). Together, these results suggested that HDAC1/2/3 all possess intrinsic histone desuccinylase activity and represent the major histone desuccinylases in mammalian cells.

### HDAC1/2/3 possess robust histone desuccinylase activity in vitro

**Fig. 4:**
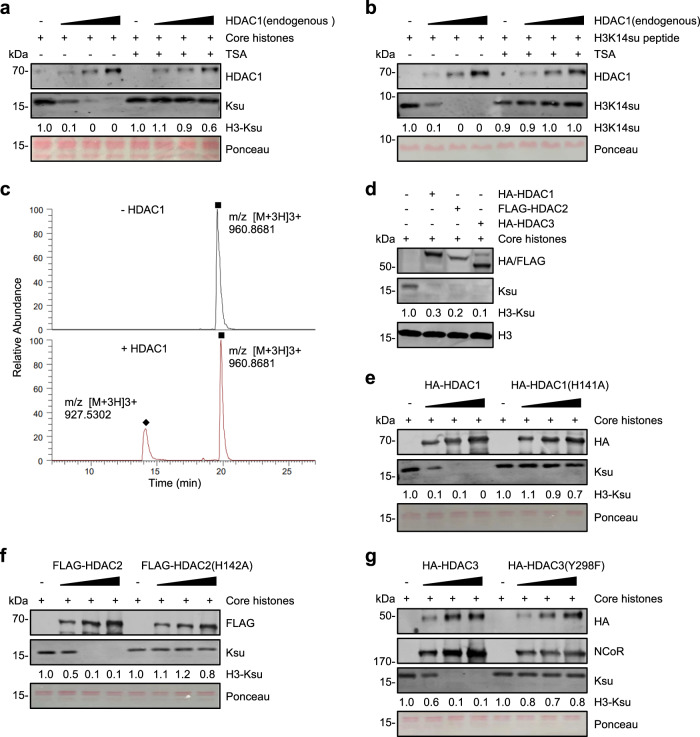
Histone desuccinylation by HDAC1/2/3 in vitro. **a**, **b** Endogenous HDAC1 proteins were immunoaffinity purified from HeLa nuclear extracts by magnetic bead-conjugated HDAC1 antibody and then used for in vitro desuccinylation assay using either histone substrates (**a**) or synthetic H3K14su peptide (**b**). The increasing amount of HDAC1-associated beads is 2 μL, 4 μL, and 8 μL. Histone substrates were prepared from HeLa cells treated with 1 μM TSA for 12 h. TSA concentration in in vitro reaction was 10 μM and the reaction time was 2 h. **c** Analysis of desuccinylation of synthetic H3K14su peptide by HDAC1 by mass spectrometry analysis. Desuccinylation reaction was carried out with 2 μL HDAC1 beads and incubated for 30 min. Quadrate indicates the H3K14 succinylated peptide peak, rhombus the H3K14 unmodified peptide peaks; the succinyl (m/z 960.8681 [M + 3H]3 + ) and desuccinyl (m/z 927.5302 [M + 3H]3 + ) peptides were indicated. **d** In vitro histone desuccinylation by HA-HDAC1, FLAG-HDAC2, and HA-HDAC3 immunoaffinity purified from transfected HEK293T cells. Histone substrates were prepared from HeLa cells treated with 1 μM TSA for 12 h. **e**–**g** In vitro histone desuccinylation by HA-HDAC1 and HDAC1(H141A) mutant (**e**), FLAG-HDAC2 and HDAC2 (H142A) mutant (**f**), and HA-HDAC3 and HDAC3 (Y298F) mutant (**g**). All tagged HDAC1/2/3 and mutants were expressed and immunoaffinity purified from transfected HEK293T cells. A 2-fold increased series of HDAC1/2/3 and mutants were used in the reactions. WB analysis also showed that NCoR was copurified with HA-HDAC3, indicating that both wild-type and mutant HDAC3 were incorporated into the NCoR complex.

Using recombinant proteins, previous studies failed to detect desuccinylase activity for all HDACs in vitro^18^. We confirmed that recombinant HDAC2, HDAC3, and HDAC8 purified from bacteria were inactive in an in vitro histone descuccinylation assay, whereas recombinant SIRT5 was active (Supplementary Fig. S5). We thus surmised that the histone desuccinylase activity may require HDAC1/2/3 in their native protein complexes. In this regard, HDAC1 and HDAC2 have been shown to exist in multiple large protein complexes known as Sin3A^26,31^, Mi-2/NuRD/NURD^27,28,39^, and CoREST complexes^40^, whereas HDAC3 is the subunit of the SMRT and NCoR corepressor complexes^29,30^. To test this idea, we first used a highly specific HDAC1 antibody to purify the endogenous HDAC1 complexes from HeLa nuclear extracts via one-step immunoaffinity purification. For in vitro histone desuccinylation assay, we prepared core histone substrates by acid extraction method from HeLa cells treated with 1 μM TSA for 12 h, which markedly increased the histone succinylation level. We found that the purified HDAC1 exhibited a robust and dose-dependent histone desuccinylase activity and, furthermore, this activity could be completely blocked by the addition of TSA (Fig. 4a). Similarly, the purified HDAC1 was also highly active in desuccinylation of H3K14su from a synthetic H3 peptide substrate (Fig. 4b). We further confirmed by mass spectrometry (LC-MS) that a short incubation of the purified HDAC1 with H3K14su peptide generated a desuccinylated H3K14 product (Fig. 4c).

To more vigorously test histone desuccinylase activity of HDAC1/2/3 complexes in vitro, we ectopically expressed HA-HDAC1, FLAG-HDAC2, HA-HDAC3 and their corresponding enzymatic activity-deficient mutants in HEK293T cells. The ectopically expressed wild-type and mutant HDAC1/2/3 were then purified from the whole-cell extracts via one-step HA- or FLAG-tag based immunoaffinity purification. We surmised that a portion of ectopically expressed HDACs would be incorporated into and thus purified as the endogenous protein complexes. As shown in Fig. 4d, we found that all three HDACs were active in an in vitro histone desuccinylation assay. Subsequent detailed analysis showed that each purified HDAC exhibited a robust and dose-dependent histone desuccinylase activity, whereas the purified corresponding mutant was inactive (Fig. 4e–g). WB analysis of purified wild-type and mutant HDAC3 revealed the presence of NCoR proteins, thus confirming that at least a portion of HDAC3 and its mutant proteins were incorporated into the endogenous SMRT/NCoR protein complexes (Fig. 4g).

### HDAC1 has a broad site specificity in histone desuccinylation

Although extensive proteomic approaches have identified more and more Ksu sites in core histone proteins^8,16,22^, this far most identified histone succinylation and desuccinylation enzymes appear to be site-specific, with KAT2A succinylating H3K79 and SIRT7 desuccinylating H3K122^15,22^. Having demonstrated that HDAC1/2/3 possess robust and redundant histone desuccinylase activity, we wished to determine the histone desuccinylation site-specificity of HDAC1. As the endogenous histones had a relatively low level of histone succinylation that hindered the identification of desuccinylation by LC-MS technology, we resorted to in vitro assay using core histone substrates prepared from TSA-treated HeLa cells. In two independent sets of experiments, we compared the number of trypsin-digested, succinylated histone peptides from the reactions with and without the addition of purified HDAC1 by LC-MS analysis. As summarized in Table 1, collectively a total of 11 succinylation sites were detected in histones H2A/H3/H4 from the HDAC1 untreated samples, and 5 out of 11 Ksu sites, namely H3K18su, H3K37su, H4K5su, H4K16su, and H4K20su have not been reported before. Interestingly, although the pan-Ksu antibody detected only weak succinylation on H4, six H4Ksu sites were identified, including K5, K8, K12, K16, and K20, the sites well known for acetylation (Table 1). These results suggest that WB analysis by pan-Ksu antibody might underestimate H4 succinylation level. Also shown are the spectrum counting numbers of the succinylated peptides detected in the experiments. Notably, as compared to the mock reactions, no succinylated histone peptide was detected in the reactions with the addition of HDAC1, suggesting that HDAC1 had efficiently desuccinylated all 11 Ksu sites from histone substrates. We thus conclude that HDAC1 has a broad site-specificity in histone desuccinylation.

**Table 1:** MS analysis of in vitro histone desuccinylation by HDAC1.

Sites	Peptide sequence	Counting number − HDAC1	Counting number + HDAC1
H2AK95	R.NDEELNKsuLLGR.V	SC = 3	SC = 0
H3K18	R.KsuQLATKAAR.K	SC = 3	SC = 0
H3K23	R.KQLATKsuAAR.K	SC = 2	SC = 0
H3K37	R.KSAPATGGVKKsuPHR.Y	SC = 3	SC = 0
H3K56	R.YQKsuSTELLIR.K	SC = 2	SC = 0
H4K5	R.GKsuGGKGLGKGGAKR.H	SC = 11	SC = 0
H4K8	R.GKGGKsuGLGKGGAKR.H	SC = 17	SC = 0
H4K12	R.GKGGKGLGKsuGGAKR.H	SC = 10	SC = 0
H4K16	R.GKGGKGLGKGGAKsuR.H	SC = 7	SC = 0
H4K20	R.KsuVLRDNIQGITKPAIR.R	SC = 4	SC = 0
H4K31	R.DNIQGITKsuPAIR.R	SC = 13	SC = 0

In vitro desuccinylation reactions were performed using histone substrates prepared from TSA-treated HeLa cells and without (−) or with (+) addition of immuno-affinity-purified HDAC1. The resulting histones were analyzed for succinylated histone peptides by LC-MS. “SC” represents spectrum counting number, which is semi-quantitative for peptide abundance.

### The HDAC1 and HDAC3 minimal core complexes are highly active for histone desuccinylation in vitro

**Fig. 5:**
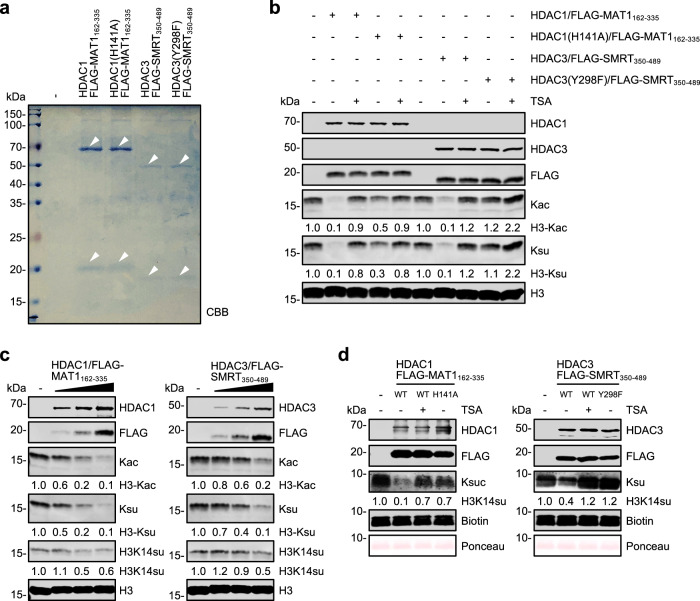
Histone desuccinylation by minimal HDAC1 and HDAC3 core complexes in vitro. **a** CBB staining showing minimal HDAC1 and HDAC3 core complexes purified from HEK293T cells. The positions of wild-type and mutant HDAC1/3, FLAG-MAT1_162–335_, and FLAG-SMRT_350–489_ are indicated by white arrows. **b** In vitro desuccinylation assay for minimal HDAC1 and HDAC3 core complexes prepared from mammalian cells. Approximately 200 ng wild-type or mutant complexes were used for in vitro assay. Histone substrates were prepared from TSA-treated HeLa cells. TSA concentration in in vitro reaction was 10 μM and the reaction time was 2 h. **c** Dose-dependent histone desuccinylation by purified wild-type HDAC1/FLAG-MAT1_162–335_ and HDAC3/FLAG-SMRT_350–489_ core complexes. The increasing amount of HDAC1 and HDAC3 core complexes: 100 ng, 200 ng, and 400 ng. **d** In vitro desuccinylation assay using synthetic H3K14su peptide substrate. Desuccinylation reactions were carried out without or with 200 ng HDAC1 or HDAC3 core complexes as indicated for 2 h. TSA concentration in in vitro reaction was 10 μM.

Previous elegant structural and functional studies demonstrated that a mammalian expressed minimal core complex consisting of HDAC3 and the deacetylase-activation domain from the SMRT corepressor is active in deacetylation^41–43^. Similarly, the minimal NuRD core complex consisting of HDAC1 and the ELM2-SANT domains from MTA1 is active in vitro^43^. To further characterize HDAC1 and HDAC3 histone desuccinylation activity, we accordingly expressed and purified the HDAC1/FLAG-MAT1_162–335_, HDAC3/FLAG-SMRT_350–489_, and the corresponding mutant HDAC1 and HDAC3 complexes from HEK293T cells by immunoaffinity purification using anti-FLAG M2 agarose beads. The complexes were then eluted from beads using 3× FLAG peptide and estimated to be at least 80% pure by Coomassie Brilliant Blue (CBB) staining after separation by SDS-PAGE (Fig. 5a). Subsequent in vitro assay revealed that the minimal wild-type HDAC1 and HDAC3 core complexes possessed a robust, TSA-sensitive histone deacetylation and desuccinylation activity (Fig. 5b, comparing lane 2 with lane 3 and lane 7 with lane 8). The residual deacetylase and desuccinylase activity observed for the HDAC1 mutant complex could be due to the presence of a small fraction of endogenous HDAC1/2/MAT1_162–335_ complex, as FLAG-MAT1_162–335_ presumably could also form a complex with endogenous HDAC1/2. Consistent with this idea, the addition of TSA blocked residual histone deacetylation and desuccinylation by the HDAC1 mutant complex (Fig. 5b, comparing lane 4 with lane 5). Enzyme dosage (Fig. 5c) and time course experiments (Supplementary Fig. S6) revealed that both HDAC1 and HDAC3 minimal core complexes catalyzed histone deacetylation and desuccinylation with comparable activity and kinetics. Furthermore, using synthetic H3K14su peptide as a substrate, we demonstrated that the wild-type HDAC1 and HDAC3 core complexes again displayed a TSA-sensitive desuccinylation activity and this activity was not detected for the mutant complexes (Fig. 5d). Thus, we concluded that the HDAC1 and HDAC3 minimal core complexes possess an intrinsic histone desuccinylase activity that is comparable to its deacetylase activity.

### HDAC8 may lack an intrinsic histone desuccinylase activity

HDAC8 is also a member of class I HDACs. Unlike HDAC1/2/3, HDAC8 does not appear to form a stable corepressor complex in cells^44^. Our test with recombinant HDAC8 prepared from bacteria failed to detect any histone desuccinylase activity in vitro (Supplementary Fig. S5d). As bacterially expressed HDAC8 might fold improperly, we expressed and purified from HEK293T cells FLAG-tagged wild-type HDAC8 and a mutant defective in HDAC activity due to the mutation of the residue Asp101 to Leu (D101L)^45^. As a positive control, we also expressed and purified from HEK293T cells FLAG-tagged wild-type HDAC2 and a mutant with the residue His142 converted to Ala (H142A) (Supplementary Fig. S7a). CBB staining showed that, as expected, FLAG-HDAC8 and HDAC8(D101L) mutant were purified essentially as a single protein, whereas FLAG-HDAC2 and FLAG-HDAC2(H142A) were co-purified with additional endogenous proteins, presumably MTA1/2/3 and RbAp46/48 proteins^27,46^ (Supplementary Fig. S7a). Subsequent in vitro desuccinlation assay using core histone substrate detected no desuccinylase activity for mammalian expressed HDAC8, whereas a robust desuccinylase activity was observed for the wild-type HDAC2 but not HDAC2(H142A) mutant (Supplementary Fig. S7b, c). Interestingly, we did observe that ectopic overexpression of the wild-type HDAC8 but not HDAC8(D101L) mutant in HEK293T cells resulted in reduced levels of histone acetylation and succinylation (Supplementary Fig. S7d). Because mammalian expressed and purified HDAC8 was inactive for histone desuccinylation in vitro, we suggest that HDAC8 may lack an intrinsic histone desuccinylase activity and may regulate histone succinylation in cells indirectly.

### Histone succinylation is highly enriched at the promoter region and positively correlates with transcriptional activity

**Fig. 6:**
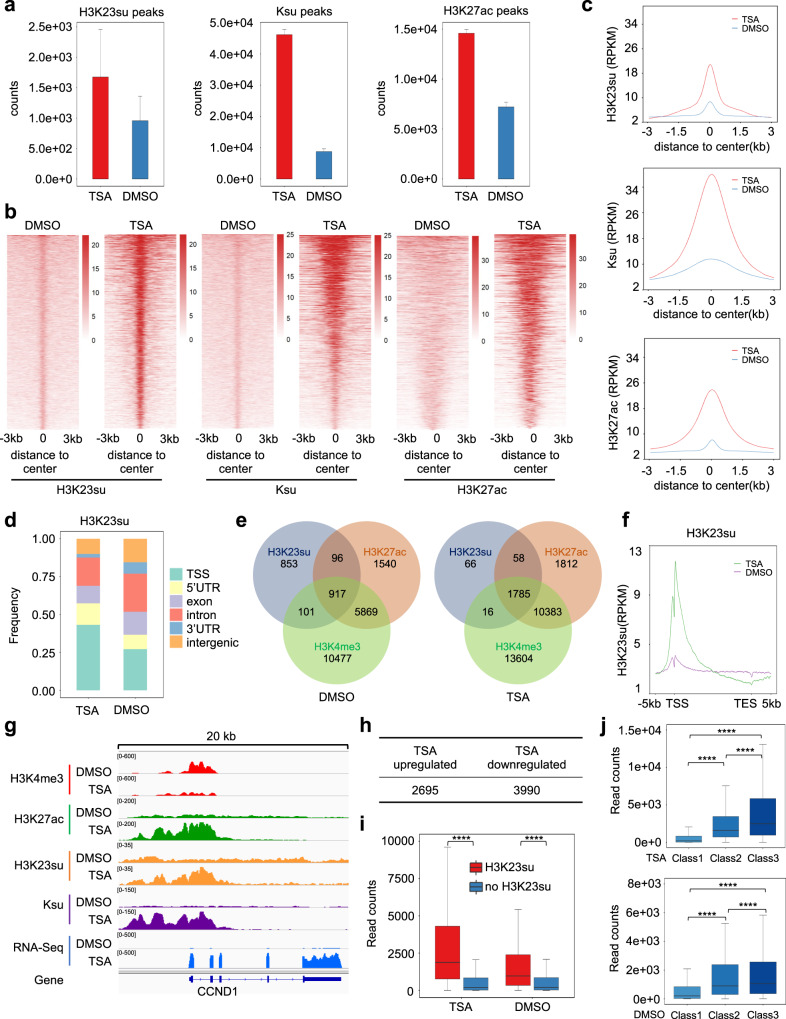
Promoter enrichment of histone succinylation and relationship with transcription. **a** Merged numbers of H3K23su, Ksu, and H3K27ac peaks detected in TSA-treated and TSA-untreated HeLa cells by ChIP-seq. ChIP-seq experiments were carried out with three independent biological samples. **b** Sorted and centered heatmaps showing peak intensities of H3K23su, Ksu, and H3K27ac in TSA-treated and TSA-untreated HeLa cells. **c** The average plot showing that TSA treatment led to markedly increased intensities of H3K23su, Ksu, and H3K27ac peaks. **d** Genomic feature distribution of H3K23su peaks. The relative proportions of H3K23su peaks in the TSS, 5’UTR, exons, introns, 3’UTR, and intergenic regions in TSA-treated and TSA-untreated HeLa cells are displayed. **e** Venn diagram showing the number of H3K23su peaks that show co-occupancy with H3K4me3 peaks and H3K27ac peaks in TSA-treated and TSA-untreated cells. **f** Meta gene analysis showing H3K23su occupancy profiles in TSA-treated and TSA-untreated cells. Note that TSA treatment drastically elevated H3K23su TSS occupancy. **g** IGV browser snapshots showing the distribution of reads around the TSS of the actively transcribed *CCND1* gene for the indicated histone modifications and RNA-seq. **h** Number of genes that were upregulated or downregulated by TSA treatment detected by RNA-seq analysis. **i** Box plots comparing the expression levels of genes with and without H3K23su peaks in their TSS regions in control (DMSO) treated cells. Integrated ChIP-seq and RNA-seq data analyses were carried out with three independent biological replicates. **j** Box plots showing the relationships between H3K23su peak intensity at TSSs and levels of transcription. Three groups of genes were categorized according to the levels of H3K23su peak intensity at TSSs.

To assess the genomic landscape of histone succinylation, we performed chromatin immunoprecipitation followed by high-throughput DNA sequencing (ChIP-seq) by using pan-Ksu and H3K23su antibodies in both control and TSA-treated HeLa cells. We also carried out ChIP-seq for H3K27ac, a marker for both promoter and enhancer. Three biological ChIP-seq replicates were of high quality (Supplementary Fig. S8) and were merged for data analysis. Consistent with observed increased histone succinylation upon TSA treatment, the numbers of H3K23su and Ksu peaks were significantly increased in TSA-treated cells as compared to the DMSO-treated control cells (Fig. 6a). Furthermore, sorted and centered heatmaps showed that both H3K23su and Ksu peak intensity also substantially increased upon TSA treatment (Fig. 6b). The marked difference in peak numbers detected for H3K23su and Ksu likely reflected the difference in antigen-binding affinity of the antibodies. As expected, both the number and intensity of H3K27ac peaks were significantly increased in TSA-treated cells (Fig. 6a, b). The drastic stimulatory effect of TSA on histone succinylation was well illustrated by the average peak read plot shown in Fig. 6c. When H3K23su and Ksu peaks were allocated according to genomic feature, we observed that both H3K23su and Ksu peaks were significantly enriched at the promoter region, with 27.17% H3K23su peaks in control and 43.2% in TSA-treated cells mapped to the transcription start sites (TSSs) (Fig. 6c, d), whereas 24.1% Ksu in control and 34.4% Ksu peaks in TSA-treated cells were mapped to the TSSs (Supplementary Fig. S9a). To further analyze the relationship between H3K27su peaks and promoters, we downloaded ChIP-seq data for H3K4me3, a marker for TSS, from a published study^47^. In accordance with the promoter enrichment, we found that 52% of H3K23su peaks in the control cells overlapped with H3K4me3 peaks and the co-occupancy rate increased to a remarkable 94% in TSA-treated cells (Fig. 6e), whereas 40% of Ksu peaks in the control cells overlapped with H3K4me3 peaks and the co-occupancy rate increased to 54% in TSA-treated cells (Supplementary Fig. S9b). Meta gene analysis further demonstrated that H3K23su is overwhelmingly enriched at the TSS region, especially after TSA treatment (Fig. 6f). To virtually illustrate the genomic landscape of histone succinylation, we selected two genomic regions, e.g., *CCND1* locus (Fig. 6g) and *ILF3* locus (Supplementary Fig. S9c), in which H3K23su and Ksu peaks co-occupy with H3K4me3 and H3K27ac peaks at the TSS regions and TSA treatment robustly elevated their levels. These results indicate that histone succinylation is preferentially targeted to the TSS regions of actively transcribed genes. Furthermore, as TSA treatment drastically increased promoter histone succinylation, HDAC1/2/3 must actively desuccinylate histones to sustain a physiological chromatin landscape in the promoter region.

To investigate how histone succinylation may regulate transcription, we also carried out RNA-sequencing (RNA-seq) analysis comparing transcription profiles in control and TSA-treated cells from three biological replicates, which had excellent quality and reproducibility (Supplementary Fig. S9d–f). This analysis revealed that TSA treatment resulted in 2695 upregulated (log_2_(FoldChange) > 1, P.adj < 0.05) and 3990 downregulated (log_2_(FoldChange) < –1, P.adj < 0.05) genes (Fig. 6h and Supplementary Fig. S9f). To better define the relationship between histone succinylation and transcription, we integrated H3K23su ChIP-seq and RNA-seq data in both TSA-untreated and TSA-treated cells. First, we found that in both TSA-treated and TSA-untreated cells genes with H3K23su peak at TSSs exhibited a much higher transcription level than the ones without H3K23su peak (Fig. 6i). Second, when the genes with H3K23su peaks at TSSs were subdivided into three classes according to the levels of H3K23su peaks, we observed a trend of positive correlation between the level of H3K23su and transcription (Fig. 6j). Thus, our data suggest that histone succinylation in promoters contributes to transcriptional activation.

## Discussion

Growing evidence designates lysine succinylation, which features a bulky negative moiety, as a prevailing post-translational modification with critical roles in various biological processes including metabolism and transcription^2,9,48–50^. While SIRT5 has been recognized as the key desuccinylase responsible for mitochondrial protein desuccinylation^5,15,16,20,21,51^, little is known about the enzyme(s) responsible for histone desuccinylation. In this study, we demonstrated that HDAC1/2/3 rather than the SIRT family proteins are the major histone desuccinylases that play a critical role in restraining histone succinylation in the promoter region.

Previous structural analysis revealed a unique mode of interaction between SIRT5 and negatively charged Ksu residue that differs from the interaction between HDACs and Kac residue^5^. Consistent with this notion, multiple in vitro studies failed to detect desuccinylase activity for recombinant HDACs^18^. We confirmed bacterially expressed HDAC2, HDAC3, and HDCA8 were inactive in histone desuccinylation in vitro (Supplementary Fig. S5). Although SIRT5 is primarily a mitochondrial protein, SIRT7 was shown to desuccinylate H3K122su both in vitro and in vivo, and loss of SIRT7 resulted in aberrant chromatin compaction and genome instability^22^. Together, these findings have led to the general conclusion that desuccinylation is most likely exerted by the SIRT family deacetylases. However, using a pan-Ksu antibody as a tool, we observed that inhibition of SIRT family deacetylases had little effect on histone succinylation, whereas inhibition of HDACs by TSA and SAHA or class I HDACs by MS275 resulted in a marked increase of histone succinylation (Figs. 1 and 2). We showed that combinatorial KO or KD of HDAC1/2/3 resulted in marked elevation of histone succinylation, whereas KO of SIRT5 had no effect (Fig. 3 and Supplementary Fig. S2). We further showed that ectopic expression of HDAC1, HDAC2 or HDAC3 but not their HDAC activity-deficient mutants resulted in a global reduction of histone succinylation in cells (Fig. 3). Moreover, we showed that immunoaffinity-purified endogenous HDAC1 complex, ectopically expressed and immunoaffinity-purified HDAC1/2/3 but not their corresponding mutants, and the highly purified minimal HDAC1/MAT1 and HDAC3/SMRT core complexes but not their corresponding mutant complexes were highly active in histone desuccninylation in vitro (Figs. 4 and 5). In fact, the highly purified HDAC1/MAT1 and HDAC3/SMRT minimal core complexes exhibited comparable histone desuccinylation and deacetylation activities in our in vitro assays (Fig. 5c and Supplementary Fig. S6) and were also active in desuccinylation of synthetic H3K14su peptide (Fig. 5d). Together, these results provide compelling evidence that HDAC1/2/3 possess intrinsic histone desuccinylase activity. It is noteworthy that, although ectopic expression of wild-type HDAC8 but not a HDAC activity-deficient mutant downregulated histone succinylation in cells, both bacterial- and mammalian-expressed HDAC8 proteins were inactive in our in vitro histone desuccinylation assay (Supplementary Fig. S7), suggesting that HDAC8 may lack an intrinsic histone desuccinylase activity and may regulate cellular histone succinylation indirectly. Previous studies have demonstrated that HDAC1/2/3 catalyze not only histone deacetylation, depropionylation, and debutyrylation, but also histone decrotonylation^32^, de-β-hydroxybutyrylation^33^ and delactylation^34^. Our finding that the HDAC1/2/3 are also the major histone desuccinylases further expands their substrate repository and defines them as the major and versatile histone deacetylases. Given that HDAC1/2 exist in multiple distinct corepressor complexes in cells, it is necessary in future to address how different HDAC1/2-containing corepressor complexes behave in histone desuccinylation. In addition, it is critical to define in the future through structural study how HDAC1/2/3 minimal complexes can accommodate succinylated moiety and catalyze histone desuccinylation.

Our in vitro desuccinylation assay coupled with mass spectrometric analysis revealed 11 histone Ksu sites that were desuccinylated by HDAC1 (Table 1). Interestingly, while WB analysis using pan-Ksu indicated that H3 is the most prominently succinylated histone, mass spectrometry identified more Ksu sites and peptides from H4 (Table 1), suggesting that the pan-Ksu antibody may bias the detection of succinylated H3. All 11 identified Ksu sites were effectively desuccinylated by HDAC1 in vitro, suggesting that these sites are bona fide substrates for HDAC1 desuccinylation. It is noteworthy that the histone substrates used in our assay were prepared from TSA-treated HeLa cells, the detected Ksu sites in the mock desuccinylation reactions, therefore, most likely represent the histone desuccinylation sites catalyzed by HDAC1/2/3 in cells. It is also noteworthy that H2BK120su and H3K122su are not altered by TSA treatment and KO or KD of HDAC1/2/3, implying that these sites are likely controlled by SIRT family proteins. Consistently, SIRT7 has been shown to catalyze H3K122su desuccinylation^22^. Our transcriptome analysis revealed that SIRT7 KO had minimal effect on transcription, whereas KO of HDAC1/2/3, as expected, resulted in substantial changes of transcription with many genes upregulated (Supplementary Fig. S10). Thus, our study suggests that while HDAC1/2/3 and SIRT7 (possibly other SIRTs) likely catalyze desuccinylation on distinct histone sites, HDAC1/2/3 are likely the major enzymes that control the transcriptional impact of histone succinylation.

Although previous studies have shown enrichment of histone H3K79su and H3K122su on the promoters of active genes^15,16^, we were surprised by the overwhelming enrichment of H3K23su on the TSS regions, especially in TSA-treated cells (Fig. 6f). This raises an interesting question as to the succinyltransferase(s) involved in selective promoter succinylation. Although KAT2A was shown to be enriched at the promoter, it is known only for H3K79 succinylation^15^. As H3K23su peaks are highly overlapped with H3K27ac peaks, we suggest that CBP/p300 could be the candidates for bulk histone succinylation on the promoters. Consistent with its net change of charge and bulkier side chain, histone succinylation has been shown to reduce nucleosome stability and strongly stimulate transcription in vitro^8,11,14^. Thus, we speculate that promoter-oriented histone succinylation is likely to facilitate chromatin remodeling to allow the assembly of RNA polymerase transcription machinery at the promoter. Given that TSA treatment results in marked elevation of histone succinylation in the promoter, we surmise that HDAC1/2/3 are constantly desuccinylating histones on the active promoters to maintain transcription homeostasis. In this regard, HDAC1/2/3 are known to be enriched at actively transcribed genes in mammalian cells^52^. In support of this notion, analysis of available ChIP-seq ENCODE data revealed substantial enrichment of HDAC1 and HDAC2 in gene promoters (Supplementary Fig. S11). Furthermore, multiple lines of evidence indicate that succinyl-CoA is relatively abundant in the nuclear compartment^7^ and lysine succinylation is a prevalent post-translational modification on histones^2^. Our finding that HDAC1/2/3 are the major histone desuccinylases provides novel insight into the dynamics of histone succinylation and paves a path to further elucidate the biological and pathological functions of histone succinylation.

## Materials and methods

### Cell lines, antibodies, and reagents

Human cervical cancer cell line HeLa, human colon carcinoma cell line HCT116, human breast cancer cell lines MCF7 and MDA-MB468 were cultured in Dulbecco’s modified Eagle’s medium (DMEM) (Gibco), and human breast cancer cell line SUM159 was cultured in Ham’s F12 nutrient medium (F12) (Gibco). All cells were cultured in medium supplemented with 10% FBS, 100 U/mL penicillin, and 100 mg/mL streptomycin in 5% CO_2_ at 37 °C. The following antibodies were used in this study: pan-Ksu mouse mAb (PTM-Biolabs 419), pan-Ksu rabbit pAb (PTM-Biolabs 401), H2BK120su (PTM-Biolabs 409), H3K14su (PTM-Biolabs 421), H3K23su (PTM-Biolabs 422), H3K122su (PTM-Biolabs 413), SIRT5 (Cell Signaling Technology D8C3), and other antibodies as described^32^. TSA and SAHA were purchased from Selleck, NAM from Beyotime, and sodium butyrate from Sigma.

### Plasmids

The plasmids of HA-HDAC1, FLAG-HDAC2, HA-HDAC3, and all mutants were as described^32^. Plasmids for KO of *HDAC1/HDAC2/HDAC3* and *SIRT5/7* in HeLa cells were constructed by cloning the guide RNA encoding DNAs into the lentiviral CRISPR-Cas9-V2 vector, with the following guide RNAs:

sg*HDAC1*-1: TTCGGTGAGGCTTCATTGGG

sg*HDAC1*-2: GGATTCGGTGAGGCTTCATT

sg*HDAC2*-1: TGGGTCATGCGGATTCTATG

sg*HDAC2*-2: GATGTATCAACCTAGTGCTG

sg*HDAC2*-3: TACAACAGATCGTGTAATGA

sg*HDAC3*-1: TTCCCTCTAGGTACCACCCT

sg*HDAC3*-2: TCCCTCTAGGTACCACCCTC

sg*SIRT5*-1: GATTTCACTCTGTTTAGGTA

sg*SIRT5*-2: AAGCACATAGTCATCATCTC

sg*SIRT7*-1: CGCAGGTGTCGCGCATCCTG

sg*SIRT7*-2: GCGTCTATCCCAGACTACCG

sg*SIRT7*-3: AAATACTTGGTCGTCTACAC

### KO with CRISPR-Cas9-V2 sgRNA or KD with siRNA

CRISPR-Cas9-V2 sgRNA plasmids for target genes or vector were transfected into HeLa cells with LipoFiter (Hanbio) according to the manufacturer’s instructions, and 48 h after infection, puromycin was added at a final concentration of 1 μg/μL to select for transfected cells. After selection with puromycin for 72 h, the cells were collected for WB analysis (for HDAC1/2/3) or isolation of KO cell lines derived from single cell cultures (SIRT5). KD of HDAC1/2/3 individually or in combination with siRNAs was performed according to the Genepharma gene manufacturer’s instruction with RNA Lipofiter (Hanbio), and cells were harvested 72 h after transfection for WB analysis. The sequence of siRNA targeting *HDAC1/HDAC2/HDAC3* were listed below:

si*HDAC1*(sense 5’-3’): GCCUGUGAGGAAGAGUUCUCCGAUU

si*HDAC2*(sense 5’-3’): UCUAACAGUCAAAGGUCAUGCUAAA

si*HDAC3*(sense 5’-3’): CGGGAUGGCAUUGAUGACCAGAGUU

### Histone preparation by acid extraction

Core histones were purified from HeLa cells using a standard acid extraction protocol as described^53^. Briefly, collect cultured 5E6 cells and re-suspend cell pellet in 1 mL hypotonic lysis buffer (10 mM Tris-Cl, pH 8.0, 1 mM KCl, 1.5 mM MgCl_2_ and 1 mM DTT), and incubate for 30 min on a rotator at 4 °C to promote hypotonic swelling of cells and lysing by mechanical shearing during rotation. Pellet the intact nuclei by spinning in cooled tabletop centrifuge: 10,000× *g* for 10 min at 4 °C. Entirely discard supernatant with pipette and re-suspend nuclei in 400 µL of 0.4 N H_2_SO_4_. Incubate on a rotator for at least 30 min or overnight. Spin samples in cooled tabletop centrifuge to remove nuclear debris: 16,000× *g* for 10 min. Add 132 µL TCA dropwise to histone solution and invert the tube several times to mix the solutions (final concentration of TCA is 33%). Incubate the solution on ice for 30 min. Pellet histones by spinning in cooled tabletop centrifuge: 16,000× *g* for 10 min at 4 °C. Carefully remove supernatant with pipette and wash histone pellet with ice-cold acetone without disturbing it. Spin in microcentrifuge 16,000× *g* for 5 min at 4 °C. Carefully remove all of the supernatant with pipette and air-dry histone pellet for 20 min at room temperature. Dissolve histone pellet in an appropriate volume of ddH_2_O (typically 100 µL, scale with quantity of cellular source).

### WB analysis and IF staining

WB analysis and IF staining were performed as described^32^. For WB analysis, whole-cell extracts were prepared by lysing cells directly in 1× SDS loading buffer and histones were prepared as above. Protein samples were separated by SDS-PAGE (8% for total proteins and 15% for histones) and transferred onto polyvinylidene fluoride (PVDF) membranes. After blocking with 7% non-fat milk in PBST, membranes were incubated with the primary antibody at 4 °C overnight. After 3 times washing with PBST and incubation with the appropriate secondary antibody, the membranes were analyzed using an Odyssey infrared imaging system (LI-COR Biosciences).

For IF staining, cells in 48-well plate were washed with PBS (137 mM NaCl, 2.7 mM KCl, 10 mM Na_2_HPO_4_, 2 mM KH_2_PO_4_) prior to fixation in 4% paraformaldehyde at room temperature for 30 min. Cells were then incubated with 1% Triton X-100 on ice for 15 min, blocked with 5% BSA (in PBST) at 37 °C for 60 min, incubated with antibody at 37 °C for 2 h, washed three times with PBST, and followed by incubation with secondary antibody against mouse or rabbit IgG. Images were acquired with an Olympus microscope system.

### Preparation of bacterially expressed recombinant HDAC2/3/8 and SIRT5 proteins

For purification of recombinant HDAC2/3/8 and SIRT5 proteins, GST-tagged HDAC2/HDAC3/HDAC8/SIRT5 proteins were induced in *Escherichia coli* with 1 mM IPTG at 16 °C overnight. For expression of hSMRT_350–489_ and mNCoR_390–498_ to form the minimal HDAC3/SMRT and HDAC3/NCoR complexes, GST-tagged hSMRT_350–489_ or mNCoR_390-–498_ were expressed in *E. coli* as above. All proteins were purified by GST affinity column and obtained after thrombin cleavage and concentrated using the Amicon Ultra 10K or 50K Centrifuge Filter Devices (Millipore).

### Immunoaffinity purification of endogenous HDAC1 complexes, ectopically expressed HDAC1/2/3/8, HDAC1/FLAG-MAT1_162–335_ complex, and HDAC3/FLAG-SMRT_350–489_ complex

To purify native endogenous HDAC1 complexes for in vitro desuccinylation assay, 0.5 mL HeLa nuclear extracts (10 mg/mL proteins) were incubated with rotation with magnetic bead-conjugated HDAC1 antibody (5 μg anti-HDAC1 antibody in 30 μL beads) for 4 h. After extensive wash with PBS and a final wash with desuccinylation buffer, the bead-associated HDAC1 complexes (2 μL, 4 μL, and 8 μL beads) were used for in vitro desuccinylation reaction as shown in Fig. 4a, b. For the preparation of ectopically expressed HDAC1/2/3/8 proteins from HEK293 cells, plasmids encoding *HA-HDAC1*, *FLAG-HDAC2*, *HA-HDAC3*, *FLAG-HDAC8* and their corresponding enzymatic defective mutants were transfected individually into HEK293 cells. The transfected cells were cultured in suspension for 72 h and harvested. Cells were lysed with the triple volume of IP lysis buffer (25 mM Tris-HCl, pH 8.0, 150 mM NaCl, 1% Triton X-100, 1 mM EDTA, 10% Glycerol, 1× protease inhibitor cocktail, 1 mM DTT) on a rotator at 4 °C for 30 min, and the supernatants were prepared after centrifugation at 12,000× *g*, 4 °C for 20 min. For preparation of HDAC1/FLAG-MAT1_162–335_, HDAC3/FLAG-SMRT_350–489_, and their corresponding HDAC mutant complexes from mammalian cells, untagged HDAC1 or HDAC1(H141A) mutant was co-transfected and expressed with FLAG-MAT1_162–335_, whereas untagged HDAC3 or HDAC3(Y298F) mutant was co-transfected and expressed with FLAG-SMRT_350–489_, in HEK293T cells for 48 h. One-step affinity purification of the resulting HDAC complexes using anti-FLAG M2 beads was performed and eluted with 3× FLAG peptide as described^53^. The resulting HDAC or complexes were examined for purity by SDS-PAGE followed by CBB staining.

### In vitro histone desuccinylation assay

In vitro histone desuccinylation assays were carried out at 37 °C for 2 h in 20 μL histone deacylation buffer (25 mM Tris-HCl, pH 8.0, 150 mM NaCl, 2 mM MgCl_2_, 1 μM Zn^2+^, 1 mM DTT) containing different amounts of bead-associated endogenous HDAC1 or approximately 100–500 ng of purified, bead-associated HA-tagged HDACs, or eluted FLAG-tagged HDAC proteins. 1 μg histones prepared from TSA-treated HeLa cells or 0.2 μg synthetic H3 peptide substrates were used as substrates. For dose-response experiments, 100 ng, 200 ng, 400 ng purified protein complexes were incubated with 1 μg core histone substrates respectively for 2 h at 37 °C. For time-course experiments, the reactions were carried out for 0 h, 1 h, 2 h, and 4 h as indicated. Histone deacetylation and desuccinylation were then evaluated by WB or mass spectrometry analysis.

### Identification of histone succinylation by mass spectrometry

Identification of succinylation sites on histones was as described^54^. Briefly, 2 μg of histones were incubated with 1 μL of propionic anhydride in 10 μL of 50 mM ammonium bicarbonate buffer (pH 8) at 37 °C for 1 h. After that, 1 μL of ethanolamine was added to quench the reaction. The mixture was digested overnight with trypsin (enzyme/protein of ∼1:50). The peptides were desalted with a C18 tip, dried under decreased vacuum, and re-dissolved in 0.1% formic acid.

The raw data were searched against the UniProt human database (20376 entries) using Mascot 2.3. Trypsin/P was selected as the digestive enzyme, and four missed cleavages sites were allowed. The mass tolerance of precursor and fragment ions was set at 10 ppm and 0.05 Da, respectively.

### Mass spectrometric identification of the synthetic H3K14su peptides

The H3 peptides in mock and desuccinylation reactions were desalted by a C18 tip and analyzed on an EASY-nLC 1200 UHPLC system (ThermoFisher Scientific) coupled to a Q Exactive HF-X mass spectrometer (ThermoFisher Scientific). The peptides were separated on a self-packed 75 μm ID capillary column (ReproSil-Pur C18-AQ, 1.9 μm; Dr. Maisch GmbH) with a length of 20 cm over a 30 min gradient of 5%–90% HPLC buffer B (0.1% FA in 80% ACN). Full mass scans were acquired in the *m*/*z* range of 300–1350 with a mass resolution of 60,000. The 12 most intensive ions were fragmented with 28% normalized collision energy and tandem mass spectra were acquired with a mass resolution of 30,000.

### Mass spectrometric identification of histone peptides

The histone peptides were desalted by a C18 tip and analyzed on an EASY-nLC 1200 UHPLC system (ThermoFisher Scientific) coupled to a Q Exactive HF-X mass spectrometer (ThermoFisher Scientific). The peptides were separated on a self-packed 75 μm ID capillary column (ReproSil-Pur C18-AQ, 1.9 μm; Dr. Maisch GmbH) with a length of 20 cm over a 30 min gradient of 5%–90% HPLC buffer B (0.1% FA in 80% ACN). Full mass scans were acquired in the *m*/*z* range of 300–1350 with a mass resolution of 60,000. The 12 most intensive ions were fragmented with 28% normalized collision energy and tandem mass spectra were acquired with a mass resolution of 30,000.

### ChIP-seq

ChIP assays were performed as previously described^55^. All ChIP-seq experiments were carried out with three independent biological samples. ChIP-seq raw reads were trimmed by Trim Galore v.0.6.5 (https://www.bioinformatics.babraham.ac.uk/projects/trim_galore/) to remove adapter sequences and poor-quality nucleotides. The trimmed reads were mapped to the human genome (assembly hg38) using Bowtie v.2.4.5^56^ with default parameters. The output SAM files were converted to binary (BAM) format. All BAM files were sorted and indexed using samtools v.1.7^57^. Samtools was also used to additionally remove nonuniquely mapped reads, as well as reads with a sequencing quality score *q* < 20. PCR duplicates were removed using Picard Tools MarkDuplicates (http://broadinstitute.github.io/picard/). BigWig files were generated using deeptools bamCoverage^58^ with --bs 60 and –normalize Using RPKM options. Peak calling was performed using MACS v.2.2.7^59^ with default parameters for Ksu, H3K23su, and H3K4me3 except for “-g hs -p 0.05 --nomodel --keep-dup all.” For H3K27ac data, MACS2 was run using the following parameters “-g hs -p 0.05 --nomodel --broad --broad-cutoff 0.1 --keep-dup all.” From the resulting peaks, those located in ENCODE black listed regions and mitochondrial DNA were filtered out, as were peaks that did not meet either the significance threshold of *q*-value < 0.01 for narrow peak calling or the significance threshold of *q*-value < 0.001 for broad peak calling. Peaks were annotated relative to genomic features using the Bioconductor package ChIPseeker^60^. The transcript database used for the annotation is “TxDb.Hsapiens.UCSC.hg38.knownGene.” We used a threshold of ±3 kb distance from the TSS of a gene for promoter annotation. Metaplots and signal heatmaps centered around peaks were generated with deepTools compiteMatrix and R package ggplot2. Box plots were also generated using the ggplot2. Venn diagrams were generated using the Intervene venn v.0.6.5^61^.

### RNA-seq analysis

For RNA-seq analysis, an equivalent number (5 × 10^6^) of DMSO/TSA-treated, control, *HDAC1/2/3* KO or *SIRT7* KO HeLa cells were used for total RNA preparation using Trizol reagent and phenol-chloroform-isopropanol extraction. Library preparation and sequencing were performed by Illumina Hiseq 2500 platform with three independent biological replicates. RNA-seq reads were aligned to the human genome (assembly hg38) using Hisat2 v.2.2.1^62^. with default parameters. FeatureCounts v.2.0.1^63^ was used to generate a matrix of mapped fragments per RefSeq annotated gene, with annotations from Gencodev26. Read counts have been normalized across samples with the median-of-ratios method proposed by Anders and Huber^64^, to make these counts comparable between samples.

### Supplementary information

Supplementary Fig. S1-11

## Data Availability

We have deposited the raw sequencing data in the Gene Expression Omnibus (GEO) with accession number GSE234006.
