# Supplementary material for: HDAC1/2/3 are major histone desuccinylases critical for promoter desuccinylation
Source: Cell Discov. 2023 Aug 15;9:85. doi: 10.1038/s41421-023-00573-9 (PMC10425439; doi:10.1038/s41421-023-00573-9)
Supplement: Supplementary file 1 — Supplementary Fig. S1-11 [file 41421_2023_573_MOESM1_ESM.pdf]

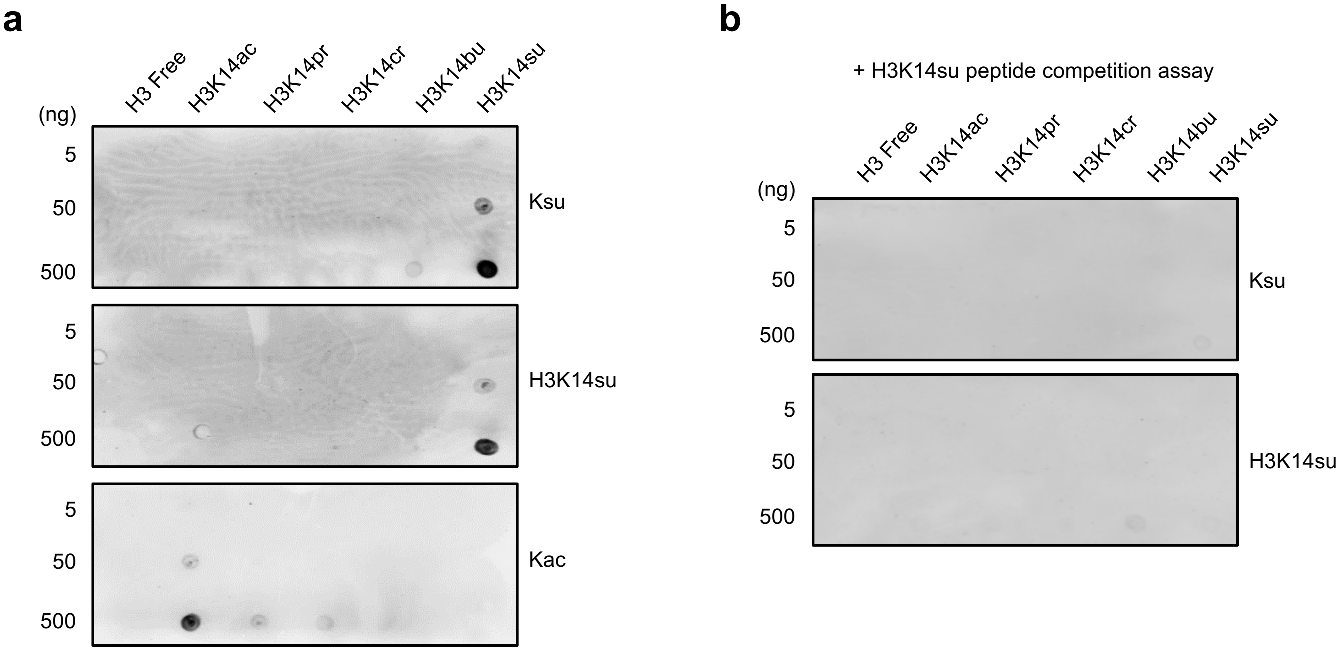

Supplementary Fig. S1. Characterization of Ksu antibody specificity by dot blots. a Dot blot was performed to evaluate the specificity of anti-pan Ksu and H3K14su-specific antibodies using various acylated H3K14 synthetic peptides. The results indicated that both Ksu and H3K14su antibodies specifically recognized H3K14 succinylated peptide but not other acylated peptides. **b** Dot blot was performed as in (**a**) with the addition of free H3K14su peptide (50 ng/ml). The results indicated that addition of free H3K14su peptide competitively blocked the detection of H3K14su peptide in dot blot.

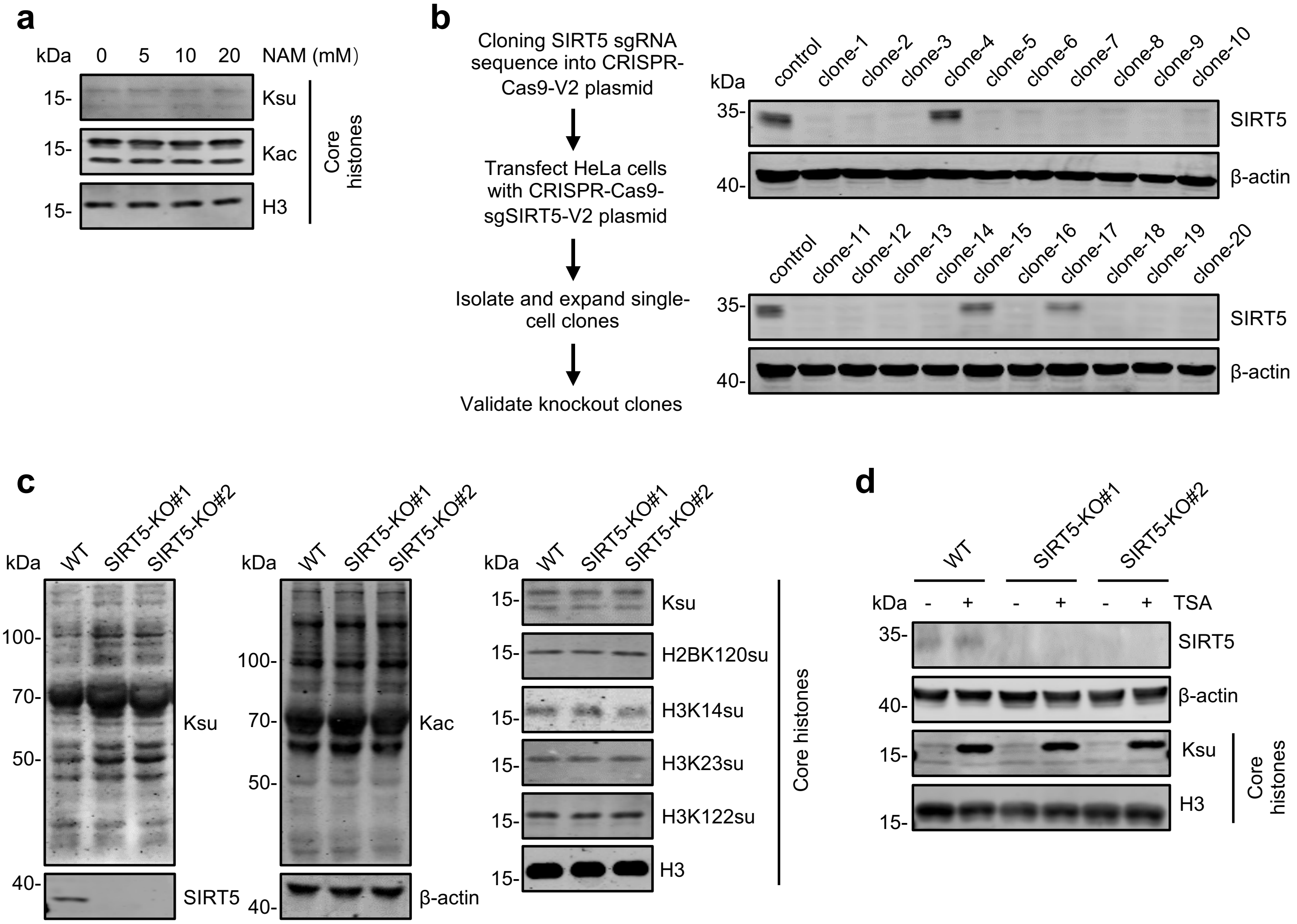

Supplementary Fig. S2. Inhibition of SIRT family deacetylases or knockout of SIRT5 has no obvious effect on histone succinylation. a WB analysis of histone succinylation and acetylation. HeLa cells were treated with an increasing concentration of SIRT inhibitor NAM for 24 h before harvested for histone preparation and WB analysis using antibodies as indicated. **b** Schematic illustration of generation of SIRT5 knockout cell lines by CRISPR-Cas9. The levels of SIRT5 in 20 single cell clones derived from CRISPR-Cas9-sgSIRT5-V2 plasmid-transfected HeLa cells were analyzed by WB. **c** WB analysis of the status of non-histone and histone protein succinylation and acetylation in control and SIRT5 knockout HeLa cells. Whole cell lysates were used for WB analysis of non-histone proteins, whereas acid-extracted histones were used for analysis of histone proteins. Two independent SIRT5 KO HeLa cell lines were used. **d** WB analysis of histone succinylation in control and SIRT5 KO HeLa cells treated with or without 1 μM TSA for 12 h.

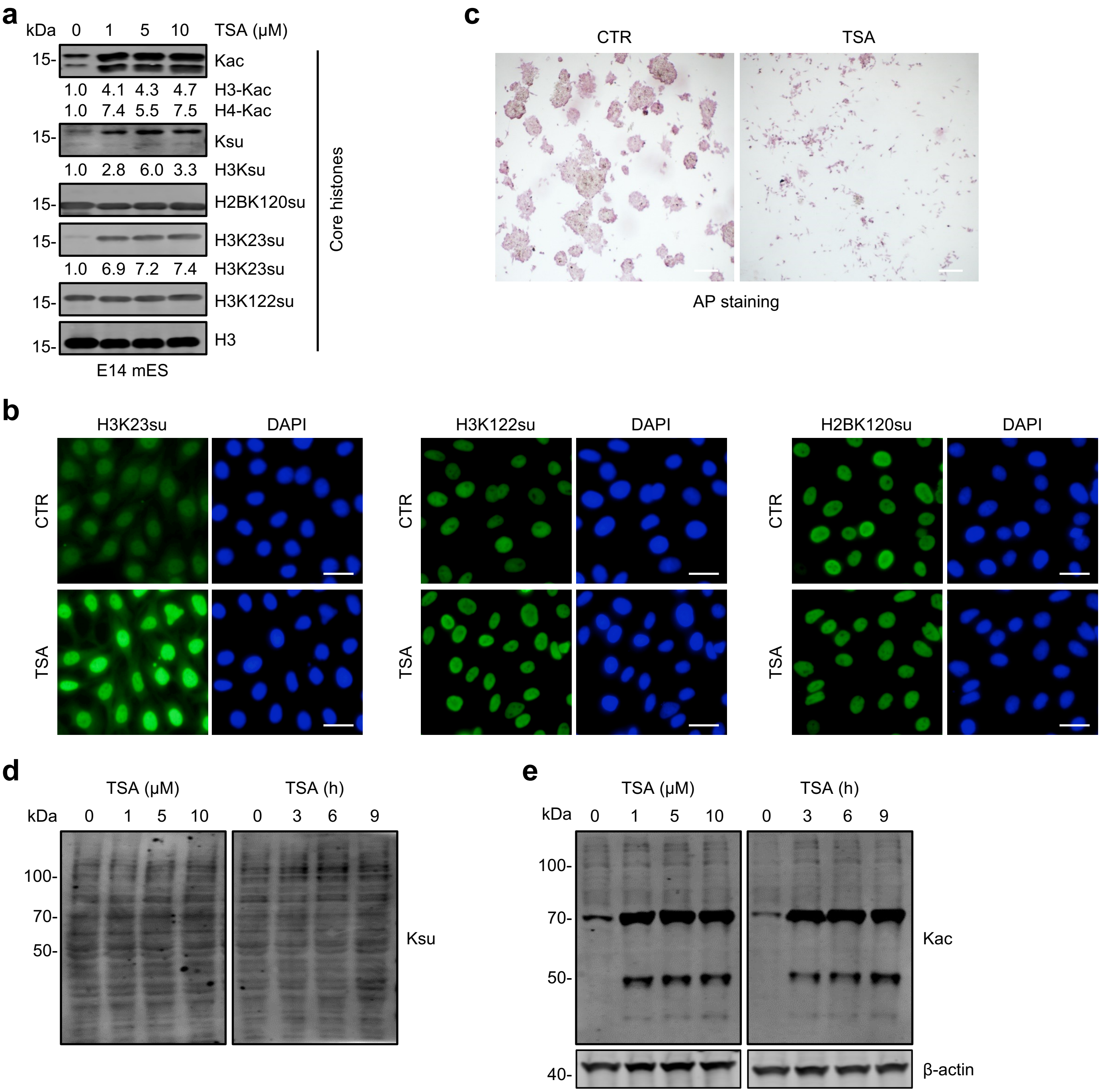

Supplementary Fig. S3. Inhibition of HDACs induced histone succinylation but had relatively minor effect on non-histone succinylation. **a** WB analysis showing that TSA treatment induced histone succinylation in mouse E14 ES cells. E14 ES cells were treated with different concentrations of TSA for 12 h before harvested for preparation of core histones and WB analysis using antibodies as indicated. Like the results observed in HeLa, HCT116 and MCF7 cells, TSA treatment elevated succinylation on H3K23 but did not appear to affect succinylation on H3K122 and H2BK120. **b** IF staining using histone succinylation site-specific antibodies confirmed that TSA treatment elevated H3K23su and had no effect on H3K122su and H2BK120su. HeLa cells were treated with 1 μM TSA for 12 h before IF staining. Scale bar, 20μm. **c** AP (alkaline phosphatase) staining showing TSA treatment (1 μM TSA for 12 h) impaired the self-renewal activity of E14 cells. Scale bar, 100 μm. **d-e** WB analysis of whole cell lysates derived from HeLa cells treated with different concentrations of TSA or 1 μM TSA for various times using pan-Ksu (**d**) or pan-Kac antibody (**e**).

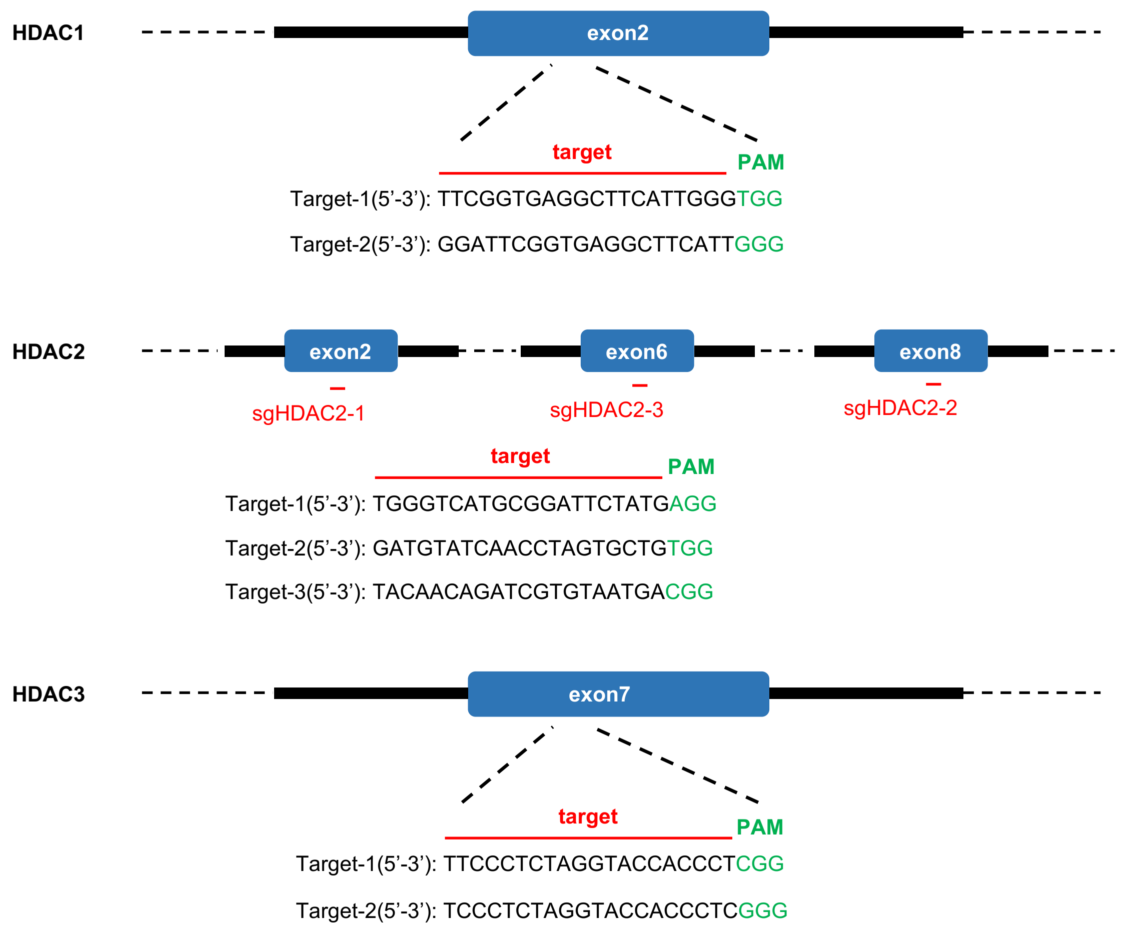

Supplementary Fig. S4. Generation of HDAC1, HDAC2, and HDAC3 knockout HeLa cells by CRISPR-Cas9. The diagram illustrates specific guide RNA sequences used for knockout of HDAC1, HDAC2, or HDAC3 genes. Also indicated are the specific exons targeted by corresponding guide RNAs. The experiments were carried as illustrated in Supplementary Fig. S2b but without isolation of single cell clones.

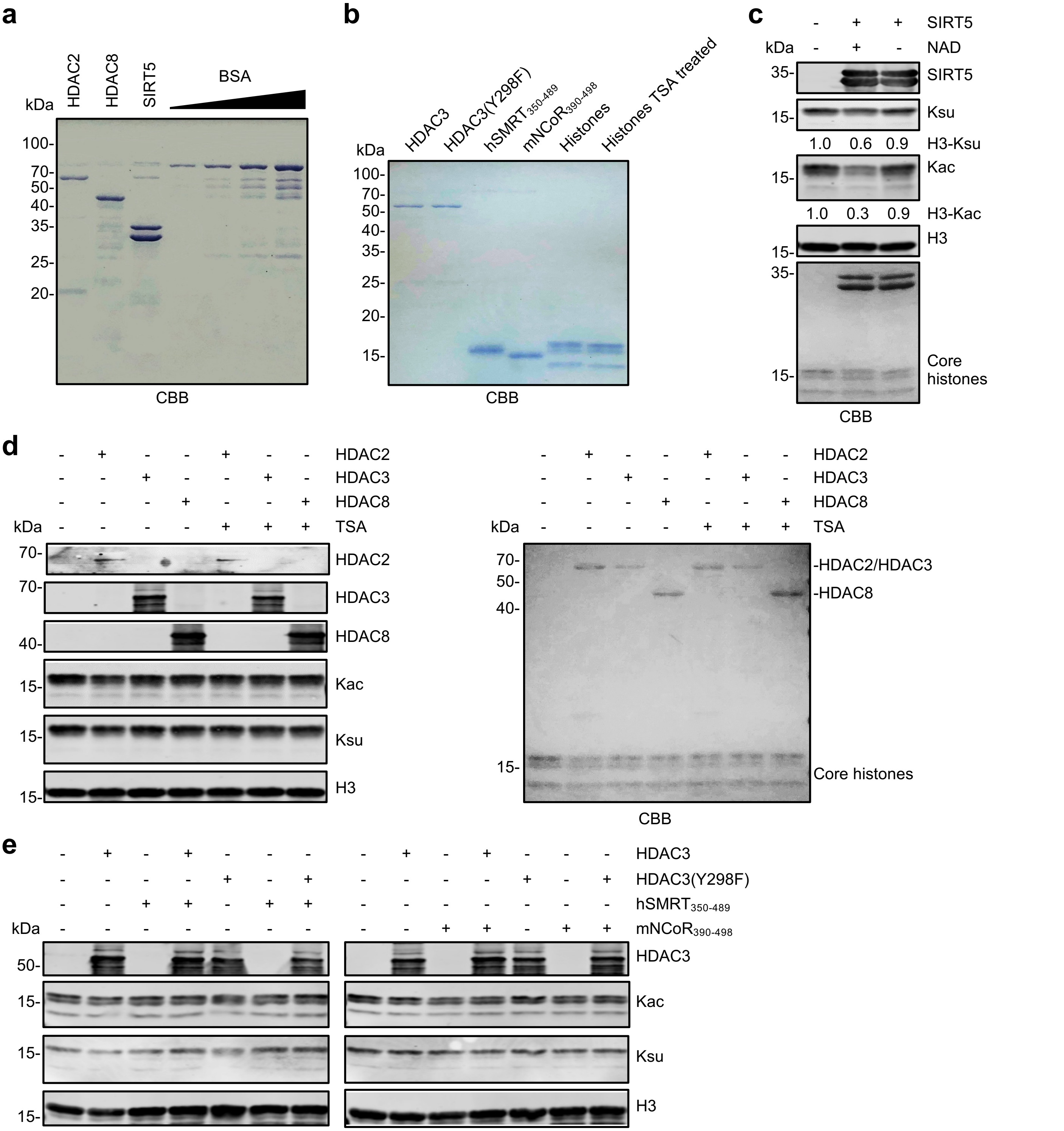

Supplementary Fig. S5. Lack of histone desuccinylase activity for bacterial expressed HDAC2, HDAC3 and HDAC8. a-b Coomassie Brilliant Blue (CBB) staining of bacterially expressed and purified HDAC2, HADC8, and SIRT5 (**a**), and HDAC3, human SMRT, and mouse NCoR fragments (**b**). **c** *In vitro* deacetylation and desuccinylation assays confirmed both activities for recombinant SIRT5. **d** *In vitro* assays revealed lack of deacetylation and desuccinylation activity for recombinant HDAC2, HDAC3, and HDAC8. **e** *In vitro* assays showed lack of desuccinylation activity for recombinant HDAC3 even in the presence of recombinant $hSMRT_{350\text{-}489}$ or $mNCoR_{390\text{-}498}$.

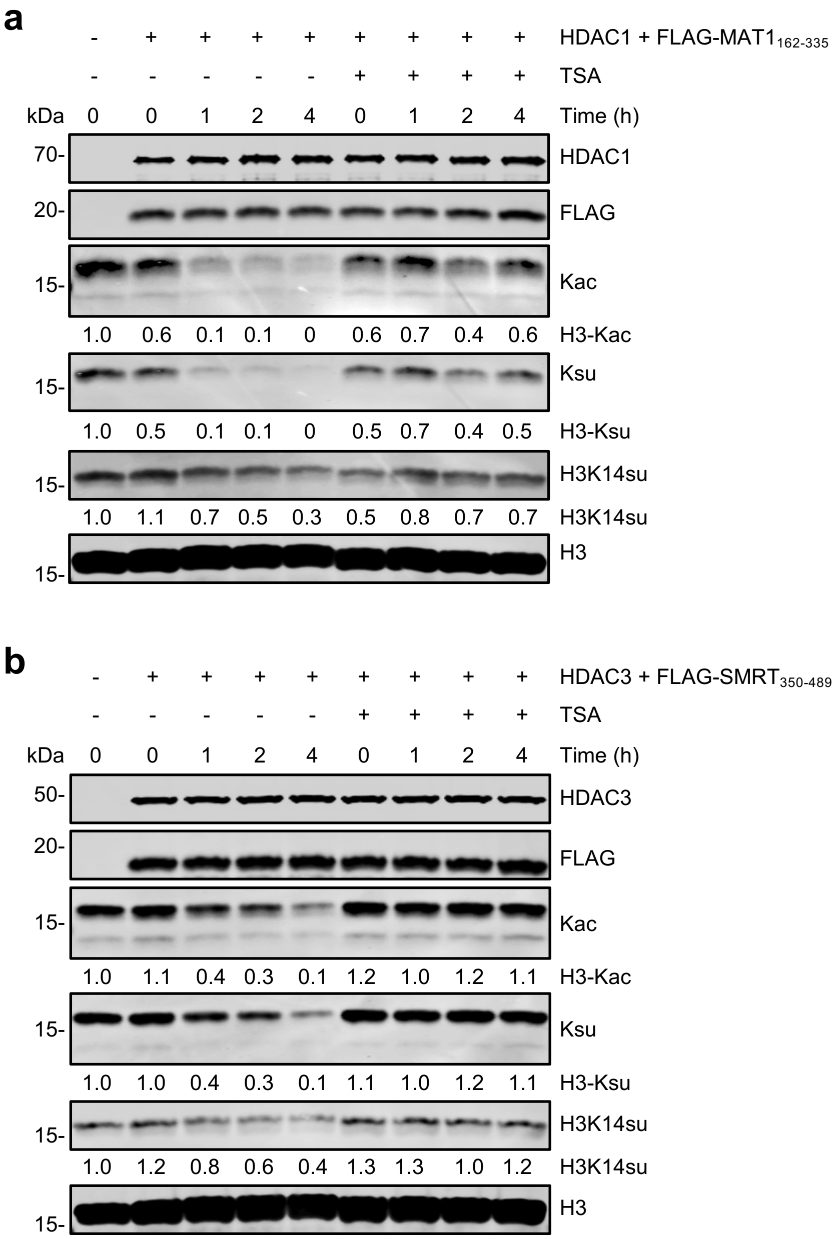

Supplementary Fig. S6. The HDAC1 and HDAC3 minimal core complexes prepared from mammalian cells displayed robust histone desuccinylation activity in time course experiments. a-b *In vitro* deacetylation and desuccinylation assays were performed with purified HDAC1/FLAG-MAT1$_{162\text{-}335}$ complex (**a**) or HDAC3/FLAG-SMRT$_{350\text{-}489}$ complex (**b**) as shown in Fig. 5a. The reactions were carried out for 0, 1, 2, and 4 h without or with addition of 10 μM TSA as indicated. Histone deacetylation and desuccinylation were then evaluated by WB analysis.

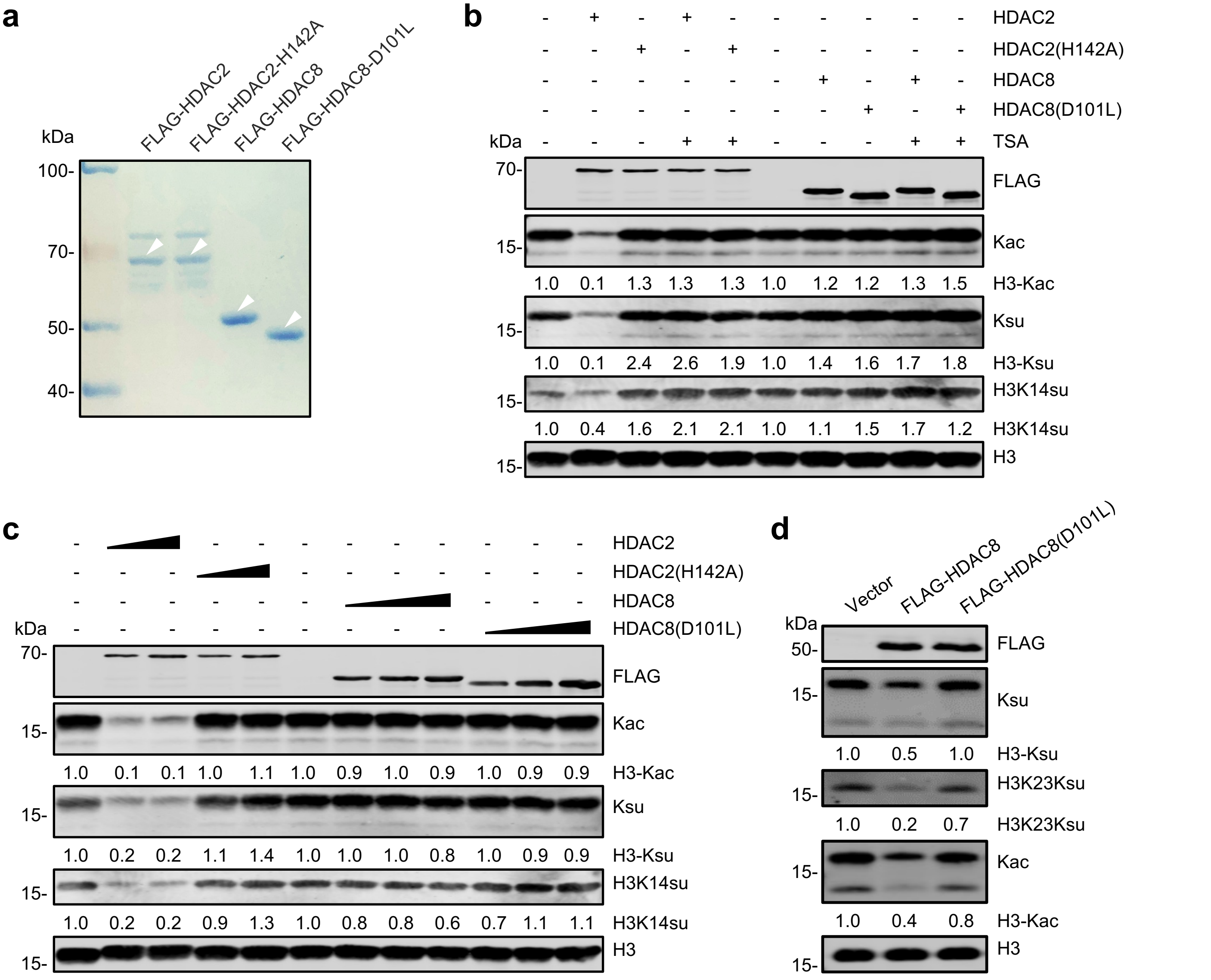

Supplementary Fig. S7. Lack of in vitro histone desuccinylase activity for HDAC8 prepared from mammalian cells. a Coomassie Brilliant Blue staining of HEK293T cell- expressed and purified HDAC2, HADC2(H142A) mutant, HDAC8, and HDAC8(D101L) mutant. All proteins were transiently expressed in HEK293T cells, purified using anti-FLAG M2 agarose beads, and eluted by using FLAG peptide. Note that HDAC8 was essentially purified as a single protein, whereas HDAC2 was co-purified with additional proteins that were likely MAT1/2 and RBAp46/48, the components of core HDAC1/2-containing NuRD complex. White arrows marked the position of FLAG-HDAC2, FLAG-HDAC8 and their mutants. **b** In vitro deacetylation and desuccinylation assays for HDAC2 and HDAC8 purified from HEK293T cells as above. Note that wildtype HDAC2 but not HDAC2(H142A) mutant displayed robust deacetylation and desuccinylation activity in this assay. Furthermore, both HDAC2 deacetylation and desuccinylation activity could be completely blocked by addition of TSA in vitro. However, no deacetylation and desuccinylation activity was detected for HDAC8 under the same condition. **c** Lack of obvious deacetylation and desuccinylation activity for HDAC8 in in vitro assays with different doses of HDAC8. **d** WB analysis showing that ectopic overexpression of wildtype but not mutant HDAC8 resulted in reduced histone succinylation.

a

Experiment	# Reads	#Peaks
CHIP_H3K23su_DMSO_1	25,091,247	894
CHIP_H3K23su_DMSO_2	25,581,366	1,387
CHIP_H3K23su_DMSO_3	25,133,289	590
CHIP_H3K23su_TSA_1	23,852,456	2,445
CHIP_H3K23su_TSA_2	22,024,093	1,699
CHIP_H3K23su_TSA_3	22,775,919	884
CHIP_H3K27ac_DMSO_1	29,361,570	7,110
CHIP_H3K27ac_DMSO_2	29,470,277	6,769
CHIP_H3K27ac_DMSO_3	29,376,905	7,733
CHIP_H3K27ac_TSA_1	28,954,566	14,459
CHIP_H3K27ac_TSA_2	30,139,867	14,292
CHIP_H3K27ac_TSA_3	31,449,059	15,006
CHIP_Ksu_DMSO_1	24,675,963	8,067
CHIP_Ksu_DMSO_2	25,125,203	8,500
CHIP_Ksu_DMSO_3	25,062,876	9,696
CHIP_Ksu_TSA_1	25,766,599	44,709
CHIP_Ksu_TSA_2	26,736,351	47,937
CHIP_Ksu_TSA_3	24,977,982	45,874

b

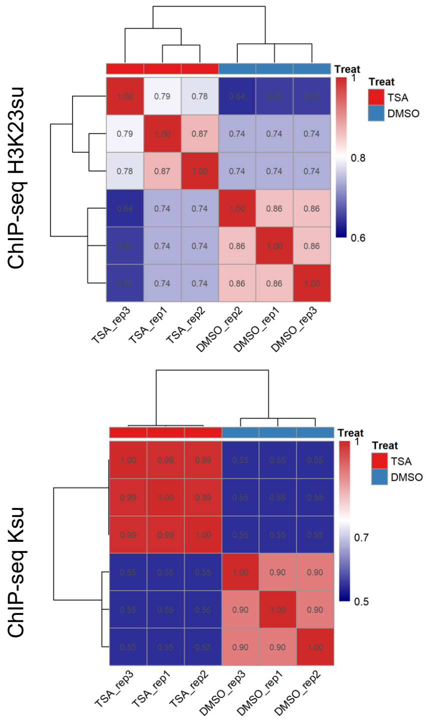

c

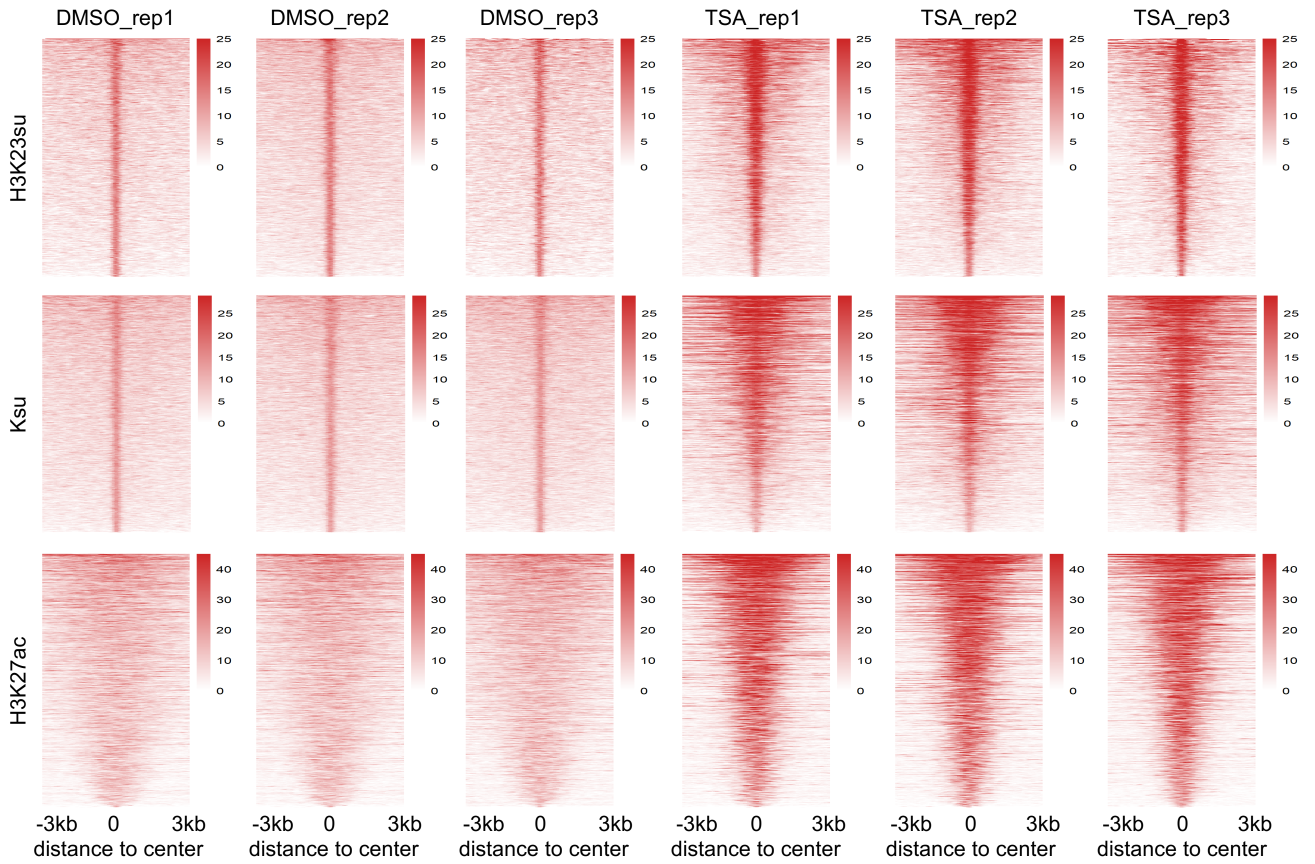

Supplementary Fig. S8. Reproducibility of ChIP-seq biological replicates. a Reads and peak numbers of the three ChIP-seq replicates of H3K23su, Ksu and H3K27ac from DMSO and TSA treated HeLa cells. **b** Heatmaps showing the Pearson's correlation coefficients of three ChIP-seq replicates of H3K23su and Ksu from DMSO and TSA treated samples. **c** Sorted and centered heatmaps of three ChIP-seq biological replicates of H3K23su, Ksu and H3K27ac from DMSO and TSA treated HeLa cells.

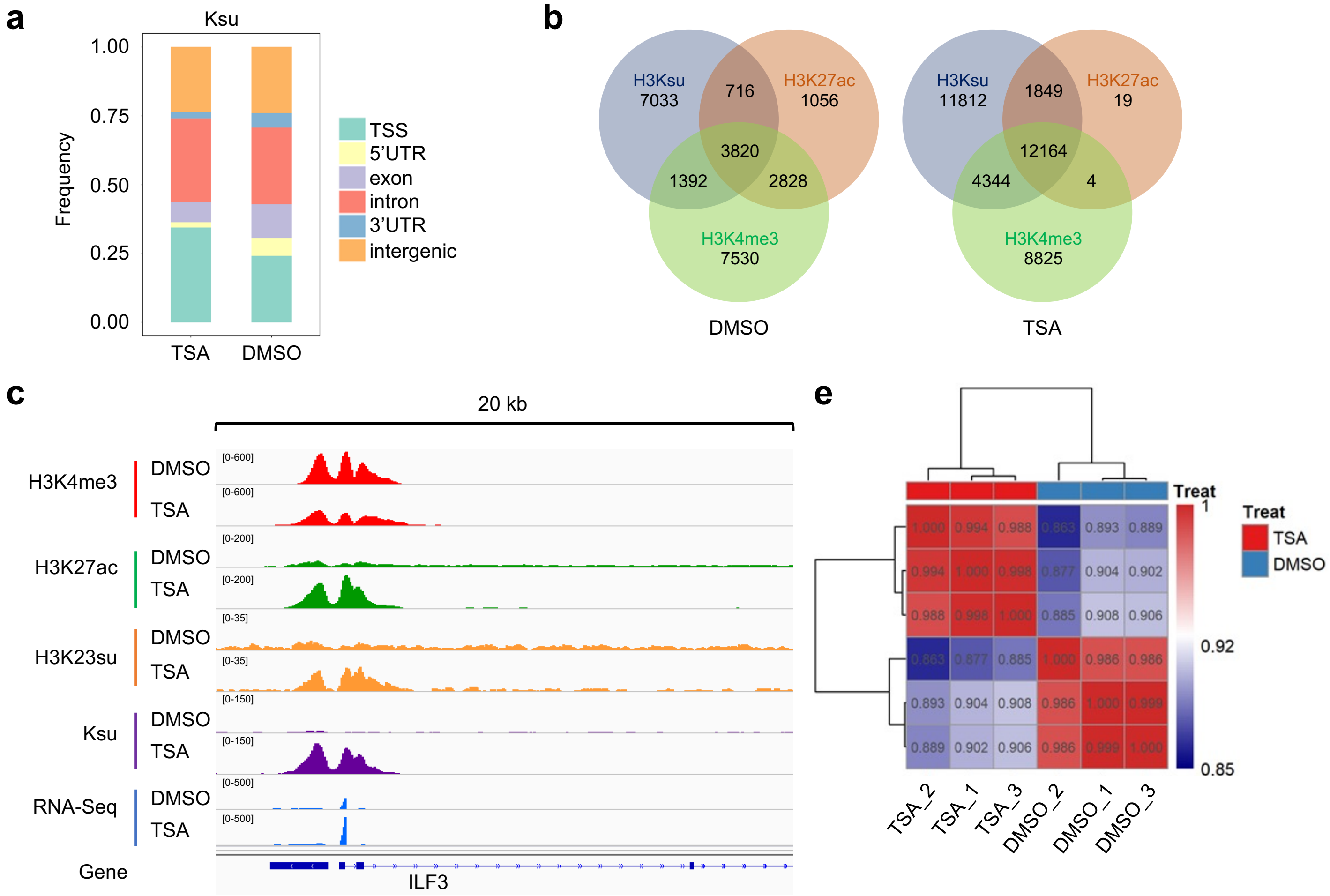

d

Experiment	Raw Reads	Mapped Reads	Mapped Ratio	Uniquely mapped Reads (MAPQ >20)	Unique ratio
DMSO_1	45,462,504	43,536,654	95.76%	40,742,584	89.61%
DMSO_2	41,723,604	39,219,011	94.00%	37,228,798	89.22%
DMSO_3	48,306,864	46,524,614	96.31%	43,788,186	90.64%
TSA_1	45,720,652	43,090,662	94.25%	39,877,286	87.21%
TSA_2	45,641,018	43,255,776	94.77%	40,400,800	88.51%
TSA_3	47,581,918	44,588,238	93.71%	41,607,546	87.44%

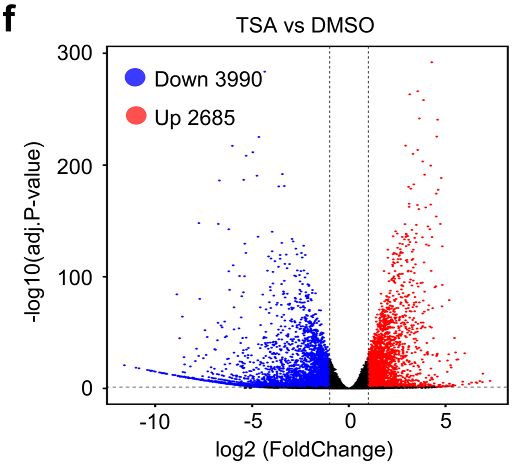

Supplementary Fig. S9. Integrated analysis of ChIP-seq and RNA-seq. a Genomic feature distribution of Ksu peaks. The relative proportion of Ksu peaks in the TSS, 5'UTR, exons, introns, 3'UTR and intergenic in TSA-treated and untreated HeLa cells were displayed. **b** Venn diagrams showing the number of Ksu peaks that show co-occupancy with H3K4me3 peaks and H3K27ac peaks in TSA-treated and untreated cells. **c** IGV browser snapshots showing the distribution of different histone modification peaks around the TSS of the actively transcribed ILF3 gene. Also shown is read peak of RNA-seq. **d** Summary of RNA-seq data. **e** Heatmap shows the Pearson's correlation coefficients of three RNA-seq replicates. **f** Volcano plot showing differentially expressed genes between DMSO and TSA treated samples, |log2(FoldChange)|< -1, p-value.adj < 0.05. RNA-seq analyses were performed in triplicates of biological repeats.

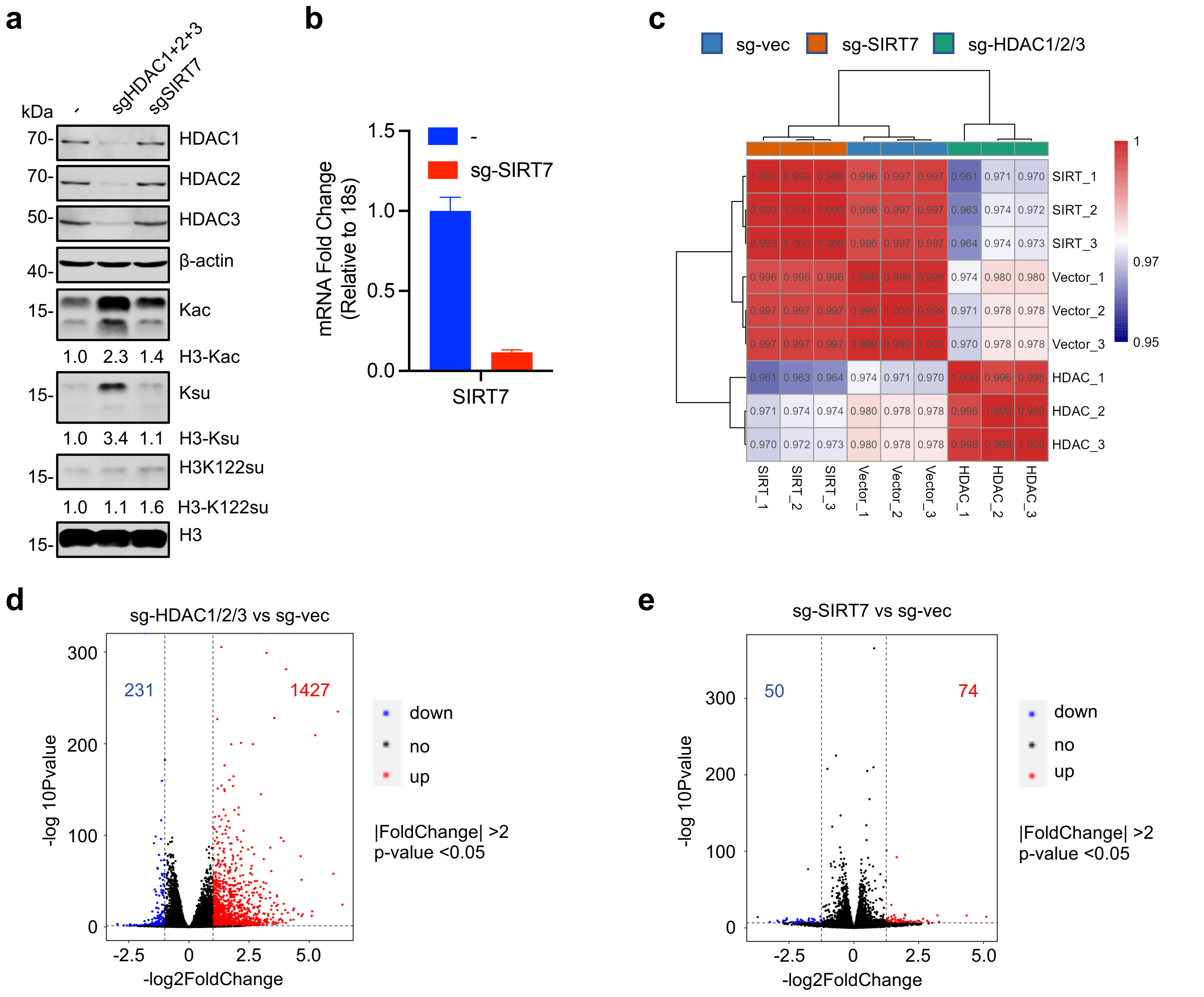

Supplementary Fig. S10. Transcriptome analysis of HDAC1/2/3 or SIRT7 knockout HeLa cells. a WB analysis of the status of histone protein succinylation and acetylation in control, HDAC1/2/3 and SIRT7 knockout HeLa cells. **b** RT-qPCR analysis showing the SIRT7 knockout efficiency in HeLa cells (SIRT7-RT-qPCR primer, forward:5'-GACCTGGTAACGGAGCTGC-3', reverse: 5'-CGACCAAGTATTTGGCGTTCC-3'). **c** Heatmap showing the Pearson's correlation coefficients of three RNA-seq replicates. **d** Volcano plot showing differentially expressed genes between HDAC1/2/3 knockout and control cells. **e** Volcano plot showing differentially expressed genes between SIRT7 knockout and control cells.

a

SRR	cell Line	TF/histone	Source
SRR11952859	MCF7	H3K122succ_AB#1	Succinylation of H3K122 destabilizes nucleosomes and enhances transcription. EMBO Rep 2021 Mar 3;22(3):e51009.
SRR11952860	MCF7	H3K122succ_AB#2	
SRR11952861	MCF7	H3K122succ_AB#3	
SRR11952858	MCF7	Input_for_H3K122succ	
SRR5459213	U251 cells	H3K79succ	KAT2A coupled with the α-KGDH complex acts as a histone H3 succinyltransferase. Nature, 2017 Dec 14;552(7684):273-277
SRR5459215	U251 cells	input	
SRR577859 SRR577860	MCF-7	HDAC2	ENCODE
SRR577916-SRR577919	MCF-7	HDAC2_input	
SRR14106126	HepG2	HDAC1_rep1	ENCODE
SRR14106127	HepG2	HDAC1_rep2	
SRR5331189-SRR5331194	HepG2	HDAC1_input_rep1	
SRR5331185 SRR5331186	HepG2	HDAC1_input_rep2	

b

Experiment	# Peak
H3K122su_AB#1	5,242
H3K122su_AB#2	5,407
H3K122su_AB#3	4,785
H3K79su	17,139
HDAC1	11,436
HDAC2	49,181

c

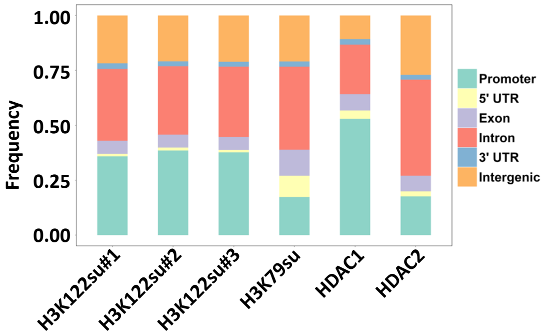

d

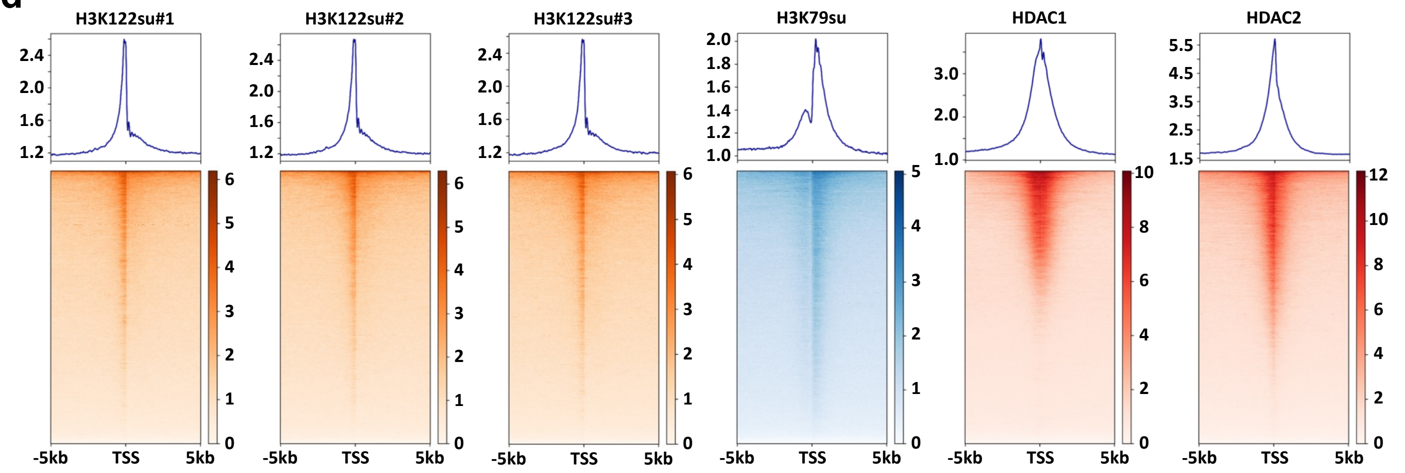

Supplementary Fig. S11. Analysis of available ChIP-seq data. a The summary of the available ChIP-seq. **b** ChIP-seq Peak numbers of H3K122su, H3K79su and HDAC1/2. **c** Genomic feature distribution of ChIP-seq peaks of H3K122su, H3K79su and HDAC1/2. **d** Sorted and centered heatmaps and the average plot show the peak intensities of H3K122su, H3K79su and HDAC1/2.
